# Genetic analysis of tolerance to combined drought and heat stress in tropical maize

**DOI:** 10.1371/journal.pone.0302272

**Published:** 2024-06-20

**Authors:** Melkamu Elmyhun, Ermias Abate, Alemu Abate, Adefris Teklewold, Abebe Menkir

**Affiliations:** 1 Department of Plant Science, College of Agriculture and Environmental Science, Bahir Dar University, Bahir Dar, Ethiopia; 2 Amhar Regional Agricultural Research Institute (ARARI), Bahir Dar, Ethiopia; 3 International Maize and Wheat Improvement Center (CIMMYT), Addis Ababa, Ethiopia; 4 International Institute of tropical Agriculture (IITA), Ibadan, Nigeria; KGUT: Graduate University of Advanced Technology, ISLAMIC REPUBLIC OF IRAN

## Abstract

Simultaneous occurrences of heat and drought stresses have a detrimental effect on growth, development and yield of maize. Heat and drought is expected to worsen maize yield losses under climate change. Selecting CDHS tolerant maize hybrids creates great opportunity for sustainable maize improvement in the tropics. The objective of current investigation was to dissect the genetic basis of CDHS tolerance in tropical maize and to determine performance of single cross hybrids under CDHS. Ninety six single-cross hybrids resulted from crossing 12 tassel blast tolerant and 12 tassel blast susceptible lines along with two *Striga* resistant commercial hybrids, a heat tolerant and a heat susceptible check hybrids were evaluated under FIRR, MDRTS and CDHS using 25x4 alpha lattice design with two replications. The results showed significant genetic variation for FIRR, MDRTS and CDHS tolerance among maize hybrids. The majority of single crosses that showed improved grain yield over their respective standard checks under MDRTS also exhibited improved grain yield over the same checks under CHDS, indicating development of CHDS tolerance hybrids. Significant and positive genotypic and phenotypic correlation of grain yield under MDRTS and CDHS implicated common genetic mechanisms controlling yield under MDRTS and CDHS. Stress tolerance indices YI, GMP, MP, HM and STI were identified as best selecting indices under both stresses. GCA variances were larger than SCA variances in each testing environment for most studied traits indicating the impotence of additive gene action than non-additive gene action to control these traits. Majority of stress indices and SCA effects demonstrated that hybrids HB18, HB41, HB91 and HB95 were high yielder under MDRTS and CDHS. Hybrids HB41, HB91 and HB95 and their parents’ scored minimum tassel blast. Parents 19 and 7 were well general combiner for grain yield and early maturity under MDRTS and CDHS indicting their valuable source of genes for hybridization. The current findings revealed that CDHS tolerance hybrids can reduce expected yield losses and maintain maize productivity in CDHS prone areas. Promising hybrids should be tested further under various drought and CHDS for commercialization.

## Introduction

Maize is a versatile multi-purpose crop playing a major role as food, feed and nutritional security in sub-Saharan Africa (SSA) where 80% of its grain is used as food [1] with more than 50% dietary energy contribution [2]. Diversified use of maize contributes to its wide cultivation in SSA on 40.7 million hectares with total grain of over 80 million tons [3] accounting 40% cereal production in Africa. In spite of its production and diverse use, maize productivity and production growth have been negatively affected by drought and heat stress [4]. One of the major factors contributing to low grain yields of maize in SSA is lack of access and slow turnover [5] of high yielding and stress resilient maize varieties.

Smallholder farmers producing maize in SSA are exposed to multiple stresses occurring simultaneously or at different phases of crop development [6]. Drought has adversely affected maize at reproductive stage by reducing successful fertilization through increased anthesis- silking interval leading to reduction of effective kernel setting [7] and finally lower yield by 90% [8]. Water requirement of maize is highly critical two weeks before and after pollination and grain filling stage, which are the most sensitive stages for water deficit [9]. Heat and drought stresses coexist in many crop production areas creating more damaging effects on plant growth and development, including yield [10, 11]. Most maize producing countries have suffered from maize yield losses due to heat stress alone and in combination with drought occurring as a result of climate change [12]. Drought, heat and combined heat and drought stress reduce maize yield by 46%, 55 and 66%, respectively, showing an additive effect of drought and heat stress on grain yield of maize [13]. Meseka et al. [14] found that 58% of maize yield lost due to drought stress alone and co-occurrence of drought and heat stress increased the loss to 77%. Extreme drought and heat stress scenarios could cause total yield loss and force farmers to abandon their farmlands [15, 16].

Since 1997, maize breeding for drought tolerance achieved considerable progress [12, 17], while organized research for heat stress tolerance was initiated in 2019 [10]. As these two stresses co-occur in farmers’ fields, finding new germplasm combining tolerance to CDHS are important to attain genetic gain in grain yield and other traits in farmers’ fields experiencing the simultaneous presence of these stresses. Consequently, combining tolerance to drought and heat stress in maize can minimize yield losses resulting from the co-occurrences of these stresses. Studies reveal that genetic improvement in tolerance to combined abiotic stresses is the most appropriate strategy for sustainable maize production [13, 18]. Understanding the genetic basis of tolerance to abiotic stresses is thus critical for selecting parental lines to develop multiple-stress tolerant hybrids and mitigate the negative effects of simultaneous presence of stresses in farmers’ fields [19, 20].

Developing stress resilient maize hybrids depends on the extent of genetic variation in the breeding material, and elucidation of stress adaptive traits inheritance [8]. Identifying superior hybrids requires GCA and SCA estimates of parental lines [10, 21]. Nasser et al. [10] explained the existence of significant GCA and SCA for grain yield and other recorded traits, with the importance of GCA effects over SCA under CDHS and well watered environment, indicating additive gene action controlling these traits. They also identified two stable and better yielding hybrids across experiments. However, Elmyhun et al. [22] reported the importance of both additive and non-additive gene actions for inheritance of most traits measured under optimum condition with preponderance of non-additive genetic effects mainly governed the traits. Oyekunle et al. [23] found larger SCA variances compared to GCA variances under drought and optimum growing conditions, underlining the manifestation of the non-additive gene action controlling most recorded traits. Annor et al. [24] argued that these contradictory results of combining ability studies on stresses tolerance could arise from differences in the genetic backgrounds of the inbred lines used, highlighting the importance of understanding the combining abilities of new stress tolerant maize inbred lines. Even though smallholder farmers’ fields are often exposed to the simultaneous presence of drought and heat stress [12], the mode of inheritance of resilience to combined stresses is rarely studied. Mittler [25] emphasize the need to understand the undesirable effect of combinations of stresses on plant growth and development to mitigate the negative impact of climate change. The present study was therefore conducted to (i) dissect the genetic basis of combined drought and heat stress tolerance in selected tropical maize and (ii) determine the effects of tassel blasting on performance of hybrids, and (iii) assess agronomic performance of the single cross hybrids under managed drought stress and full irrigation, and combined heat and drought stress.

## Materials and methods

### Genetic materials

Ninety-two S4 lines derived from bi-parental crosses of elite drought tolerant and *Striga* resistant white parental lines plus 179 S4 lines derived from backcrosses involving temperate lines as donors of desirable traits and elite drought tolerant and *Striga* resistant lines as recipients were screened at Kadawa station in Kano State of Nigeria for tolerance to heat and drought stress between February and June in 2016. During this period, the temperature varies from 33 to 45°C that facilitated screening of the lines under combined heat and drought stress by carefully controlling water supply through furrow irrigation. Amongst these, 56 promising lines with desirable agronomic traits and minimal leaf firing that were tolerant to tassel blasting plus 21 lines that were susceptible to tassel blasting were selected for further inbreeding and evaluation in hybrid combinations. These lines were further evaluated at Kadawa station during the same period in 2017 to confirm their responses to combined heat and drought stress.

Based on the results obtained in 2016 and 2017, 12 tassel blast tolerant and 12 tassel blast susceptible lines with diverse genetic backgrounds were selected for this study (S1 Table). The 24 inbred lines were divided into 6 sets each of four inbred lines, with the inbred lines in one set used as females and crossed with the four inbred lines in another set as males following the Design II mating scheme. Each inbred line was used as a female parent in one set and as a male parent in a second set. The resulting 96 hybrids (6 sets x 16 hybrids) and four checks were evaluated under managed drought stress and full irrigation at Ikenne (https://doi.org/10.25502/kpt0-e749/d and https://doi.org/10.25502/dyce-8h10/d), as well as under combined heat and drought stress (https://doi.org/10.25502/cw1h-kw05/d) at Kadawa in Nigeria for two years.

#### Performance evaluation under combined heat and drought stress

The 100 maize hybrids arranged in 25 x 4 alpha lattice were evaluated in two replications for tolerance to combined heat and drought stress at Kadawa (11°390 N, 8°270 E, and elevation 500 m) in Kano and Katsina State in Nigeria, where temperature of 33 to 45 °C occur between February and June. The soil type at Kadawa is Regosols, with mainly sandy to clay loam texture, pH of 5.9, organic carbon 4.3 g kg^−1^, and residual nitrogen 0.24 g kg^−1^ [26]. These conditions permitted evaluation of the hybrids under CDHS through careful control of the supply of water using furrow irrigation. The flowering and grain filling periods were generally in April that coincided with negligible incidence of rainfall and relative humidity of 15% to 46% that exposed the hybrids to drought and elevated temperature. The maize hybrids were planted in mid-February in 2020 and 2021 with tasseling and silking occurring in mid-April, which is the hottest period at Kadawa. The hybrids were initially grown under well-watered conditions prior to their exposure to combined heat and drought stress. A furrow irrigation system was used to supply sufficient water to the crop every four days during the first 45 days after planting. Irrigation was withheld in mid-April when mean day temperature varied from 36 to 45°C and night temperature varied from 27 to 30 °C that coincided with flowering time of the hybrids. Twenty-one days later, irrigation supply was provided once a week to allow hybrids to fill their grains until physiological maturity.

#### Performance evaluation under managed drought stress and well-watered conditions

The 100 hybrids in this trial arranged in 25x4 alpha lattice design were also evaluated with two replications under managed drought stress and full irrigation at the IITA experiment station in Ikenne (6°53’ N, 3°42’ E, altitude of 60 m) during the 2019/2020 and 2020/2021 dry seasons. The trial was planted on 21 November in 2019 and on 18 November in 2020 in two adjacent blocks. The soil at Ikenne is eutric nitosol (FAO classification) and the station has uniform experimental fields. One of the blocks received full irrigation through a sprinkler irrigation system every week from planting until the hybrids attained physiological maturity. In the second block, drought stress was induced by withdrawing irrigation from 35 days after planting to harvesting time of the trial. Each hybrid was planted in two 4 m long row plots with 0.75 m spacing between rows and 0.25 m spacing between plants within a row. Two seeds were planted in each hill and thinned them later to one plant after emergence to attain a population density of 53,333 plants per ha. At the time of sowing, we applied 60 kg N, 60 kg P and 60 kg K ha^-1^ fertilizer with an additional 60 kg N ha^-1^ fertilizer applied four weeks later. The trial field was sprayed with gramazone and atrazine as pre-emergence herbicides at the rate of 5 liters/ha, which was followed by manual weeding to keep the trials weed-free.

#### Trait measurements

Plant height (PH) and ear height (EH) were measured on five representative plants in centimeters as the distance from the base of the plant to the base of tassel and to the node bearing the uppermost ear, respectively. Number of days to 50% anthesis (ANT) and 50% silking (SIL) were recorded when 50% of the plants in a plot started shedding pollen and produced silks, respectively. Anthesis-silking interval (ASI) was calculated as the difference between DA and DS. Plant aspect (PASP) was valued as a general appearance of the plants in a plot using a scale of 1 to 5, where 1 = uniform outstanding plants free from foliar diseases and 5 = very non-uniform poor plants with severe disease symptoms. Ear aspect (EASP) was scored as the overall appearance of the ears using a scale of 1 to 5, where 1 = clean, large, uniform and well filled ears with no insect and disease symptoms, and 5 = diseased and insect damaged, rotten, variable, poorly filled, and small ears. Husk cover (HC) was recorded on a scale of 1 (very tightly organized husks, prolonged beyond the ear tip) to 5 (ear tips loose and exposed kernels). The number of plants showing tassel blasting were counted on plot basis at grain filling stages on CDHS trials and converted to percentages. The grain moisture was determined using a moisture meter on five randomly picked representative ears per plot (measured at least two times). Additional data on barren plants (BP) were recorded for combined drought and heat stress trial and leaf death score was taken for combined drought and heat stress trials. BP was measured on a scale of 1 to 9, where 1 = few plants have no ears and 9 = more than 50% or all plants bearing no ears were recorded in each plot two weeks after flowering. Grain yield was calculated form grain weight adjusted to 15% percent moisture content.

#### Data analyses

Analysis of variance was computed for each year and years’ combination using R software [27] to generate adjusted genotype means for each measured trait under MDRTS, FIRR and CDHS conditions. Combined analyses of variance across location x year combinations, which are hereafter referred to as environments, was conducted for all traits using adjusted means measured under each management condition. In the combined analysis of variance, genotypes (hybrids and standard checks) were considered as fixed effects while environments, replications within environment and blocks within replication were considered as random effects. The variance components due to hybrids within sets were portioned into variance due to female (set), male (set) and female x male (set) interaction and the interaction of each respective variance component with environment was used to compute F tests. The variance due to environment x female x male (sets) was tested using the pooled error variance. Significant means were separated using the least significant difference (Fisher’s LSD). Main effects of female (sets) and male (sets) represent the GCA while female x male (sets) interaction represent SCA effects [28].

BLUE estimates for grain yield were determined using META-R software [29]. Pearson’s correlation coefficients were computed to determine the relationships between grain yield and its components under CDHS with grain yield and its components under MDRTS as well as grain yield and its components under CDHS with grain yield and its components under FIRR using R software. Stress tolerance indices were determined from BLUE values using the various approaches provided in S2 Table.

## Result

### Combining ability estimates under different growing conditions

Year and sets had significant effects on grain yield and most other traits recorded under CDHS, MDRTS and FIRR (Table 1). Mean squares for hybrids differed significantly for grain yield and most other traits. Hybrid x year interactions were significant for grain yield under CDHS and FIRR and only a few other traits (Table 1). Mean squares for both females (sets) and males (sets) were significant for grain yield, antheis and silking days, plant height and plant aspect recorded under CDHS, MDRTS, FIRR, and for anthesis-silking interval, husk cover and ears per plant recorded under CDHS (S3 Table). Although the mean squares for females x male (set) interactions were significant for grain yield, silking days and plant height under CDHS, MDRTS, FIRR (Table 1) and under CDHS and MDRST for a few other traits (S2 Table), they were much smaller in values than the corresponding mean squares for female (set) and male (set). The year x females (sets) and year x males (sets) interactions were significant for grain yield under the three growing conditions but for a few traits under one or two growing conditions (Table 1). The absence of significant mean squares for year x female x male interaction for grain yield and other traits under CDHS, MDRTS and FIRR indicate consistence contribution female x male interaction to performance of the single-cross hybrids.

**Table 1:** Mean squares from combined analyses for grain yield and other traits of 96 hybrids involving lines with varying resistance to tassel blast tested under CDHS, MDRTS and FIRR for two years (2020 and 2021).

Source of variation	DF	Grain yield (Kgha^-1^)			Anthesis (days)			Silking (days)			Plant height (cm)			Ear height (cm)		
		CDHS	MDRTS	FIRR	CDHS	MDRTS	FIRR	CDHS	MDRTS	FIRR	CDHS	MDRTS	FIRR	CDHS	MDRTS	FIRR
Year	1	356879627***	1483127*	911283ns	512***	114.5***	1340***	530***	95***	1408***	63697***	6336***	63823***	24019***	95ns	13209***
Rep(Year)	2	6099191***	1056498*	13093327***	24***	29***	52***	33***	33***	53***	115ns	578***	1199***	107ns	406***	564***
Block (Rep xYear)	96	6862290***	495825***	896670***	10***	6***	3***	13***	7***	3***	322***	297***	141***	173***	112***	53***
Set	5	5956558***	955537***	2507371****	14***	16***	4*	14***	14***	5***	1197***	971***	1521***	961***	224***	652***
Year x Set	5	5062839***	451953ns	930577ns	6ns	0.8ns	0.5ns	7ns	3ns	0.4ns	111ns	451***	249*	126ns	81ns	17ns
Hybrid	95	3880039***	723900***	3458217***	12***	11***	12***	72ns	11***	12***	378***	311***	511***	378***	112***	210***
Year x Hybrid	95	1728089***	281035ns	1432826***	3ns	3ns	2*	66ns	11ns	2*	178***	186***	224***	171***	78*	76***
Female (Set)	18	6046756.3***	780119***	3933168***	25***	19***	15***	30***	18***	15***	438***	377***	445***	275***	132***	175***
Male (Set)	18	5036613***	837979***	3463200***	17***	15***	13***	18***	15***	12***	239***	338***	392***	218***	142***	158***
Female x Male (Set)	54	1506140***	555764***	1671125***	2ns	5***	2ns	5*	5***	2*	102*	141*	155*	46ns	57ns	49*
Year x Female (Set)	18	1378870*	475849*	888933*	3ns	4ns	2ns	6*	4ns	2ns	77ns	227***	149*	30ns	65ns	48*
Year x Male (Set)	18	2965651***	331607ns	1760625***	4ns	4ns	1ns	4ns	5ns	2ns	56ns	291***	182***	75ns	59ns	98***
Year x Female x Male(Set)	54	1052136ns	175758ns	499814ns	3ns	2ns	1ns	4ns	3ns	1ns	81ns	91ns	96ns	53ns	71ns	49ns
R^2^		0.97	0.89	0.93	0.94	0.92	0.96	0.95	0.92	0.96		0.97	0.93		0.95	0.89
CV (%)		36.54	38.8	17.35	2.44	2.98	2.32	2.63	2.97	2.18		4.86	7.36		9.2	11.42

CDHS = combined heat and drought stress, MDRTS = managed drought stress, FIRR = full irrigation, *,**, *** Corresponding mean squares significantly different from zero at *P*<0.05, *P*<0.01 and *P*<0.001 levels, respectively, ns = no significant difference at any probability level

Partitioning the hybrids sum of squares under CDHS, MDRTS and FIRR showed that the GCA variances were larger than the corresponding SCA variances for most traits, indicating the importance of additive gene action in controlling these traits. The existence of larger GCA variances compared to SCA variances for most traits in each testing condition suggesting the presence of common genetic mechanism (additive gene action) for governing these traits at CDHS, MDRTS and FIRR.

Eight lines (Entry 7, 9, 10, 14, 18, 19, 21 and 22) recorded significant or non-significant positive GCA effects for grain yield when used both as female and male parents under CDHS (Table 2). Six of these lines combined either significant or non-significant negative GCA effects for anthesis and silking days with positive significant or non-significant GCA effects for ear placement.

**Table 2:** GCA effects for grain yield and yield related traits in 96 single crosses involving 24 lines with varying resistance to tassel blast tested under combined heat and drought stress.

Line	Set	GY		ANT		SIL		PH		EH	
		Female	Male	Female	Male	Female	Male	Female	Male	Female	Male
1	1	-77.4	68.3	-0.26	-0.57	-0.18	-0.58	-3.13	1.33	-3.35	-0.40
2	1	-459.0	-155.0	1.51*	1.12	1.42	1.35*	-2.26	0.99	0.32	0.94
3	1	-141.1	-150.6	-0.58	-0.15	-0.43	-0.45	-10.01***	-5.44*	-9.09***	-7.29*
4	1	131.4	-95.4	0.72	-0.32	0.93	-0.32	-2.15	-3.62	-2.62	-4.13
5	2	288.2	-22.4	-0.42	-0.11	-0.42	-0.28	0.99	0.37	2.33	-2.03
6	2	28.5	-90.7	0.16	0.76	0.12	1.08	2.59	-0.05	4.44	-1.64
7	2	957.1*	167.6	-1.56*	-0.72	-1.58*	-0.59	-0.53	-1.34	3.95	0.32
8	2	-619.2	-348.7	1.49*	0.89	1.84*	0.90	-8.28*	-8.40*	-7.47***	-10.36***
9	3	186.0	13.2	-0.18	0.39	-0.35	0.67	2.32	2.20	0.13	2.16
10	3	243.9	287.7	-0.37	-0.26	-0.21	-0.42	8.04*	4.61	0.38	2.16
11	3	-360.7	-112.5	-0.55	-0.95	-0.53	-0.79	10.75***	-1.17	-6.47*	-1.11
12	3	-687.0	-295.3	1.23*	0.18	1.09	0.09	-12.18***	-3.64	-5.75*	-0.94
13	4	73.5	-154.8	0.49	0.52	-0.03	0.82	-0.42	-0.66	-2.17	-1.99
14	4	444.7	429.3	-0.58	-1.06	-0.20	-1.07	6.41	4.08	7.43***	3.32
15	4	-128.9	-18.4	-0.46	-0.24	-0.37	-0.39	4.90	3.77	4.30	4.96*
16	4	-400.7	131.2	1.79*	-0.01	1.27	-0.44	2.43	2.65	0.73	3.43
17	5	-363.2	-212.8	0.69	0.91	0.50	1.09	5.95	0.12	4.27	1.76
18	5	120.1	30.8	0.10	0.13	0.07	0.25	0.94	-3.66	-3.80	5.19*
19	5	1056.3*	670.7	-3.05***	-1.9***	-3.20***	-2.30***	6.96	-0.26	6.47*	4.17
20	5	-94.1	-136.1	0.93	0.65	0.95	0.94	4.50	0.99	3.52	2.28
21	6	344.9	368.9	-1.70*	-0.96	1.58*	-1.03	5.10	5.60	4.11	3.07
22	6	448.8	245.5	-1.81*	-0.78	-1.97*	-0.85	3.49	8.49	4.75*	10.83***
23	6	-211.7	-258.6	0.97	1.29*	1.02	1.34*	0.10	-2.60	-0.13	-0.48
24	6	-779.6	-361.9	1.47*	1.19*	1.85*	0.96	-4.97	-4.35	-6.27*	-3.85

Under MDRTS, seven inbred lines (Entry 7, 9, 10, 18, 19, 21 and 22) showed either significant or non-significant positive GCA effects for grain yield when used both as female and male parents (Table 3). Only two lines amongst these (Entry 19 and 22) combined either significant or non-significant negative GCA effects for anthesis and silking days with positive significant or non-significant GCA effects for ear placement.

**Table 3:** GCA estimates for grain yield and yield related traits in 96 single crosses involving 24 lines with varying resistance to tassel blast tested under drought stress.

Line	Set	GY		ANT		SIL		EH	
		Female	Male	Female	Male	Female	Male	Female	Male
1	1	5.77	-6.16	1.06	0.73	0.92	0.89*	-1.28	-0.55
2	1	-3.10	2.02	0.82	-0.15	0.82	-0.17	1.70	-0.21
3	1	-1.26	-9.12	0.04	-0.53	-0.18	-0.52	-3.26	-0.81
4	1	-1.41	-12.23	0.09	-0.20	0.15	-0.21	-0.22	-1.11
5	2	12.67	17.26	-0.10	-0.31	0.00	-0.31	0.14	-0.42
6	2	-1.01	-1.66	0.32	0.07	0.38	0.19	3.15	0.63
7	2	11.04	1.58	-0.49	0.43	-0.44	0.30	0.24	0.11
8	2	-5.54	-17.17	0.50	1.15*	0.58	1.00	-0.93	-1.98
9	3	3.15	6.19	-0.33	0.01	-0.18	0.11	-0.42	-0.18
10	3	7.61	3.25	-0.24	0.86*	-0.23	-0.77	-0.49	0.33
11	3	-14.45	-1.07	-0.78	-0.58	-0.70	-0.56	-2.45	0.60
12	3	-18.59	-11.07	1.12*	0.51	1.08	0.44	-1.58	-0.55
13	4	4.83	-6.74	0.25	0.46	0.00	0.37	0.75	0.12
14	4	-1.54	1.06	-0.04	-0.35	0.08	-0.36	-0.20	1.23
15	4	1.47	1.16	0.28	-0.12	0.07	-0.15	0.68	0.24
16	4	0.87	6.62	-0.31	-0.54	-0.31	-0.49	0.86	0.02
17	5	-6.06	5.04	1.12*	0.30	1.04	0.22	-0.77	-0.12
18	5	6.62	1.99	0.18	0.68	0.05	0.74	-0.58	-0.03
19	5	8.59	12.48	-2.4***	-1.5*	-2.3***	-1.3*	3.65	1.79
20	5	-7.33	0.12	0.25	0.63	0.26	0.73	0.67	-0.24
21	6	1.60	10.15	-0.30	-0.26	-0.11	-0.40	1.85	0.27
22	6	3.43	12.19	-2.1***	-0.9*	-2.1***	-0.9*	2.59	2.89*
23	6	-1.02	-6.33	0.24	0.63	0.26	0.65	-0.84	0.08
24	6	-6.38	-9.56	0.76	0.64	0.78	0.52	-3.26	-2.10

Under FIRR, eight inbred lines (Entry 4, 5, 6, 9, 10, 15, 19 and 22) had either significant or non-significant positive GCA effects for grain yield when used both as female and male parents (Table 4). Amongst these, four lines (Entry 6, 15, 19 and 22) combined either significant or non-significant negative GCA effects for anthesis and silking days with positive significant or non-significant GCA effects for plant height and ear placement.

**Table 4:** GCA estimates for grain yield and yield related traits in 96 single crosses involving 24 lines with varying resistance to tassel blast tested under full irrigation.

Line	Set	GY		ANT		SIL		PH		EH	
		Female	Male	Female	Male	Female	Male	Female	Male	Female	Male
1	1	198.5	-48.2	0.31	0.48	0.33	0.32	-2.32	-3.96	-0.92	-3.13
2	1	-114.5	-159.9	1.32*	0.87*	1.38*	0.98*	-0.43	0.34	-1.39	-0.61
3	1	-70.1	-47.4	-0.35	-0.26	-0.42	-0.23	-5.16*	-3.15	-2.90	-2.17
4	1	43.4	78.1	-0.01	-0.69	-0.10	-0.55	0.84	-3.70	-0.64	-2.24
5	2	403.6*	184.9	-0.22	-0.28	-0.14	-0.23	3.35	1.52	1.25	-0.19
6	2	161.5	160.7	-0.04	-0.47	-0.09	-0.34	7.32*	4.84	5.96*	2.65
7	2	44.7	-116.5	-0.30	0.77	-0.19	0.62	1.51	-2.61	4.12	-0.74
8	2	-93.6	-202.7	0.41	0.95	0.37	0.80	-2.33	-7.23	-4.15	-5.22*
9	3	415.0*	193.9	-0.58	0.02	-0.47	0.09	-0.26	1.31	-2.75	-0.31
10	3	309.8	127.8	-0.54	-0.48	-0.43	-0.41	1.36	2.21	-0.59	-0.66
11	3	-520.6*	-73.5	0.34	-1.25*	0.28	-1.13*	-12.26***	-1.52	-5.45*	-0.20
12	3	-589.2*	-166.6	1.31*	1.05*	1.33*	0.84	-10.16*	-1.00	-4.87*	0.72
13	4	-230.7	-110.4	0.19	0.54	0.05	0.46	-3.81	0.88	-1.63	-0.80
14	4	-253.0	-107.4	-0.24	-0.37	-0.14	-0.22	1.41	1.29	1.89	2.92
15	4	144.1	54.4	-0.40	-0.08	-0.51	-0.42	5.60*	2.68	2.73	0.43
16	4	26.2	139.3	-0.13	-0.26	0.05	-0.18	1.47	0.96	0.95	2.90
17	5	-28.0	1.1	0.78	0.33	0.64	0.28	1.28	-2.15	0.46	-1.37
18	5	53.5	-20.0	0.64	1.07*	0.53	0.91*	2.00	1.29	-0.37	-0.83
19	5	117.1	184.8	-2.66***	-2.07***	-2.61***	-1.83***	8.55*	3.49	9.30***	4.65
20	5	-194.8	72.0	1.02	0.56	0.94*	0.61	-0.01	2.20	-0.25	1.84
21	6	11.0	-64.5	-0.19	-0.25	-0.27	-0.29	3.49	2.53	0.002	1.19
22	6	467.2*	186.4	-2.31***	-1.31*	-2.13***	-1.25*	8.00*	5.30	6.52***	5.52*
23	6	20.2	-159.0	0.83	0.51	0.82	0.68	-1.56	-2.27	-1.98	-2.69
24	6	-321.4	-107.3	0.83	0.62	0.74	0.47	-7.88*	-3.26	-5.29*	-1.67

Of them, five hybrids were high yielder exhibiting large significant positive SCA effects for grain yield with negative SCA effects for ANT and SIL.

Forty six hybrids displayed positive SCA effects for grain yield under CDHS (S4 Table).Ten of these hybrids recorded highest positive SCA effects (Table 5) with high BLUE values for grain yield (Table 8).

**Table 5:** Hybrids with larger SCA effects for grain yield in each testing condition, the corresponding SCA estimates for grain yield in other testing condition and SCA effects of these hybrids for yield related traits.

Code	GY			ANT			SIL			PH	
	CDHS	MDRTS	FIRR	CDHS	MDRS	FIRR	CDHS	MDRTS	FIRR	CDHS	FIRR
HB9	428.26	117.1	-1.8	-0.18	-0.1	-0.11	0.69	-0.45	-0.03	-0.37	-0.02
HB18	385.20	432.7*	395.1	-0.14	0.36	-0.04	-0.08	0.37	0.12	-0.73	0.54
HB29	47.91	29.3	747.4*	-0.09	-0.51	-0.09	-0.05	-0.58	-0.003	0.10	0.84
HB33	-7.88	517.7*	417.1	-0.04	0.25	-0.01	-0.07	0.14	-0.13	1.80	-0.61
HB41	255.67	288.8	455.6	-0.21	1.41*	-0.68	-0.15	-1.22	-0.91*	-0.36	-0.15
HB42	195.22	-21.6	111.1	0.17	0.35	0.11	0.12	0.23	0.15	1.07	0.47
HB47	235.60	517.0*	274.8	-0.32	-0.31	0.29	-0.24	-0.43	0.34	-0.31	-0.13
HB51	288.37	319.2	618.3*	-0.06	-0.32	-0.24	0.00	-0.34	-0.28	-0.46	0.21
HB55	-87.87	518.9*	705.4*	-0.03	1.39*	-0.28	-0.04	-1.19	-0.33	0.90	0.76
HB56	405.36	200.4	492.6	-0.16	0.11	-0.31	-0.15	0.24	-0.36	4.21	2.27
HB58	77.93	131.6	637.6*	-0.19	-0.5	-0.64	-0.13	-0.37	-0.85	0.86	2.49
HB63	-172.88	589.6*	-1057*	0.18	0.52	0.65	0.10	0.41	0.78*	0.11	-1.06
HB64	-645.09	493.4*	-631.7	0.24	1.39	0.13	0.18	1.42*	0.11	-4.44	-0.78
HB69	142.92	749.4***	472.2	-0.10	1.47*	-0.52	-0.06	1.39*	-0.37	0.24	2.12
HB77	-226.9	475.7*	-612.4	0.14	1.93*	-0.09	0.07	1.52*	-0.02	-0.06	-1.69
HB85	714.64	205.6	235.5	-0.27	-0.6	-0.01	-0.19	-0.54	-0.02	1.01	0.04
HB91	340.79	408.0	209.5	-0.05	1.45*	-0.21	-0.06	-1.15	-0.27	0.76	0.35
HB94	-145.7	617.7***	174.8	0.19	0.05	0.21	0.08	0.13	0.16	-1.22	-0.68
HB95	492.73	195.7	733.3*	-0.12	-0.58	-0.40	-0.04	-0.53	-0.46	-0.30	2.53

Seven of them (HB9, HB47, HB56, HB69, HB85, HB91 and HB95) had negative SCA effects for 50% anthesis and silking days, plant aspect and ear aspect (S4 Table). Under MDRTS, forty four hybrids revealed either significant or non-significant positive SCA effects for grain yield (S5 Table). Nine of these hybrids had highest significant SCA effects with large values of BLUES for grain yield. Three hybrids (HB18, HB47 and HB69) scored highest BLUE values and SCA effects for grain yield under both CDHS and MDRTS conditions. Under FIRR, forty seven hybrids were well combiners for grain yield showing either significant or non-significant positive SCA effects (S6 Table).

### Effect of tassel blasting in parents on performance in their hybrids

Minimum values of grain yield for hybrids (set) ranged from 245.15kgha^-1^ to 631.5kgha^-1^ under CDHS, 228.7kgha^-1^ to 688.3kgha^-1^ under MDRTS and 1700.6kgha^-1^ to 3065.5kgha^-1^ under FIRR (Table 6). Maximum values of grain yield for hybrids (set) varied from 3816.6kgha^-1^ to 6715.8 kgha^-1^ under CDHS, 1927.3kgha^-1^ to 2395.58kgha^-1^ under MDRTS and 5172.2g kgha^-1^ to

**Table 6:** Minimum, maximum and mean values of grain yield and two yield components under CDHS, MDRTS and FIRR.

Sets	Grain yield under CDHS (kgha^-1^)			Anthesis CDHS (days)			Silking under CDHS (days)		
	Minimum	Maximum	Mean ±SE	Minimum	Maximum	Mean ±SE	Minimum	Maximum	Mean ±SE
TBR xTBR	467.5	4068.3	1890.7 ± 304.8	64.5	71.25	68.3±0.48	66.5	73	70.5±0.48
TBR xTBR	245.1	3816.6	1781.6±233.3	65.25	72.5	68.3±0.44	68.25	77.3	70.4±0.57
TBRxTBS	304.8	5705.8	2658.8±478.6	62.75	72.5	67.4±0.69	66	75.75	69.5±0.78
TBS xTBR	631.5	4025.4	2385.9±240.2	63.5	70	67.2±0.55	65.5	73.25	69.4±0.60
TBS xTBS	274.3	4474.8	2483±341.1	65	72.25	67.9±0.52	67.8	72.3	69.5±0.36
TBS xTBS	418.6	6715.8	2875.3±451.3	63	70.5	63±0.64	65	73	69.4±0.62
	Grain yield under MDRTS (kgha^-1^)			Anthesis MDRTS (days)			Silking under MDRTS (days)		
TBR xTBR	453.8	1928.5	1276.5±113.2	52.8	58.3	56±0.4	55	59.5	58.1±0.4
TBR xTBR	436.9	1927.3	1043.4±116.0	51.3	57.8	54.6±0.5	53	60	56.9±0.5
TBR xTBS	688.3	2361.2	1424.6±122.8	50.3	58	55.1±0.5	52.5	60.5	57.3±0.5
TBS xTBR	228.7	2042.3	1244.8±292	51.5	58.3	54.2±0.6	53.8	60.5	56.5±0.6
TBS xTBS	497.1	2395.5	1320.7±128.8	51.5	59	54.9±0.5	53.3	60.3	56.8±0.5
TBS xTBS	735.1	2220.6	1281.2±106.7	50.8	59	54.6±0.7	51.5	60.8	56.5±0.5
	Grain yield under FIRR (kgha^-1^)			Anthesis under FIRR (days)			Silking under FIRR (days)		
TBRxTBR	2451.8	5172.2	3932.9±200.8	50.8	56	53.2±0.3	53.3	58	55.1±0.3
TBRxTBR	1700.6	5913.7	3646±296.5	50.5	55.8	52.8±0.4	52.5	57.8	54.8±0.4
TBR xTBS	3065.5	5311.5	4280.2±155.3	48.5	54	52.4±0.4	50.5	56.8	54.4±0.4
TBS xTBR	1821	5830	4107.9±292	47.3	56.3	52±0.5	49.3	58	54±0.5
TBS xTBS	2156.3	5409.2	3722.2±237.2	49.5	53.8	52.1±0.3	51.3	56	54±0.3
TBS xTBS	3037.5	5446	3921.6±172.6	65	73	69.3±0.5	50	56.5	54.1±0.5

5913.7kgha^-1^ under FIRR. Grain yield performance of hybrids (set) had means of 1781.6±233.3 kgha^-1^ to 2875.3±451.3 kgha^-1^ under CDHS, 1043.4±116.0 kgha^-1^ to 1424.6±122.8kgha^-1^ under MDRTS and 3646±296.5 kgha^-1^ to 4280.2±155.3 kgha^-1^ under FIRR. The hybrids (set) TBS x TBS (tassel blast susceptible) (set 6) recorded the largest maximum grain yield value with highest mean followed by hybrids (set) TBR (tassel blast resistance) x TBS (set 3) for both cases under CDHS. Under MDRTS, cross combination TBS x TBS (set 5) had the highest maximum value for the same trait followed by cross combination TBR x TBS (set 3). Minimum, maximum and mean values for hybrids (set) of days to % anthesis, days to % silking and plant height under CDHS were larger than values of hybrids (set) under MDRTS and FIRR. Hybrids (set) under MDRTS exhibited lager minimum, maximum and mean values for days to % anthesis and days to % silking compared to values under FIRR.

Tassel blasting scores ranged from 0.00 for the majority of parents to 1.44 for parent 16 when it was used as a female or to 1.06 for parent 23 when it was used as a male (Table 7) with mean of 0.2. Twelve heat stress tolerant parents had blast scores on average of 0.11 and 0.09 when they crossed as female and male, whereas heat stress susceptible parents had blast scores on average of 0.26 when they crossed as female and male, respectively. Seven parents (7, 10, 14, 15, 19, 21 and 22) were the highest yielding with tassel blasting scores 0.06, 0.25, 0.13, 0.06, 0.31, 0.00 and 0.00 when they combined as females or 0.00, 0.25, 0.13, 0.06, 0.13, 0.06 and 0.25 as they crossed as male, respectively. Among these parents, four high yielding hybrids HB95 (19x16) (5393.2 kgha^-1^), HB43 (7X19) (5023.7kgha^-1^), HB85 (17x14) (4939.1kgha^-1^) and HB41 (5X19) (4771.8kgha^-1^) were generated. The remaining 10 highest-yielding hybrids were produced from parents whose yields were below the grand mean (2345.9kgha-1) and whose blast scores were below 0.5 when used as female or below 1.06 when reacted as male. The highest yielding hybrid HB95 was extracted from two heat stress susceptible parents (19 and 15) while the second highest yielding hybrid BH43 was developed from heat stress tolerant parent 7 and heat susceptible parent 19. These high yielding hybrids had low tassel blasting and leaf senescence scores as HB95 recorded 0, 2 and 2.5 scores for tassel blast, first and second leaf senescence. HB43 also scored 0, 3 and 5.5 for tassel blast, first and second leaf senescence. Better yielding hybrids had good ear and plant aspects as well as a short anthesis-silking interval, a sign of their tolerance to combined drought and heat stress.

**Table 7:** Grain yield, tassel blast and leaf senescence performance of parental lines under CDHS.

Line	GY(Kgha^-1^)		TB		FLS		SLS	
	Female	Male	Female	Male	Female	Male	Female	Male
1	1961.1	2221.1	0.12	0.06	3.63	4.75	5.88	7.25
2	1632.3	1658.8	0.00	0.06	3.00	5.13	5.63	8.00
3	1751.1	1231.0	0.12	0.00	2.88	4.38	5.38	6.75
4	2218.4	2014.9	0.06	0.06	3.25	4.50	5.75	6.88
5	2923.2	1961.9	0.06	0.13	3.25	2.50	5.50	4.88
6	2196.8	985.5	0.31	0.19	3.25	3.50	6.13	6.00
7	4462.2	3414.5	0.06	0.00	2.75	2.75	5.25	5.25
8	1053.2	1200.9	0.13	0.00	4.00	4.00	6.25	6.50
9	1702.9	2325.1	0.13	0.13	4.75	4.00	7.25	6.75
10	2763.4	2943.5	0.25	0.25	3.88	5.00	6.38	7.75
11	1753.0	2035.6	0.13	0.06	5.00	4.50	7.75	7.13
12	907.0	2239.4	0.00	0.19	5.13	3.88	7.50	6.38
13	2046.4	2265.4	0.06	0.31	4.25	3.88	6.63	6.50
14	3200.1	3743.1	0.13	0.13	3.5	4.50	6.00	7.25
15	2524.7	2787.4	0.06	0.06	3.63	3.50	6.13	5.50
16	2160.6	2705.2	1.44	0.31	3.38	3.88	6.13	6.38
17	2552.0	1848.3	0.13	0.00	4.25	3.00	7.00	5.25
18	2224.2	1999.5	0.31	0.31	4.25	4.13	6.88	6.88
19	4894.4	4470.5	0.31	0.13	3.63	3.13	6.00	5.50
20	1830.6	2317.1	0.06	0.13	3.63	3.13	5.75	5.38
21	3236.9	3696.5	0.00	0.06	4.00	3.13	6.50	5.50
22	2612.3	2925.0	0.00	0.25	4.25	3.13	7.00	5.88
23	1726.4	1780.1	0.50	1.06	4.50	3.75	7.25	6.13
24	1968.0	1530.3	0.13	0.31	4.63	4.75	7.25	7.38
Mean	2345.88	2345.85	0.1875	0.1746	3.86	3.87	6.38	6.38
LSD	601.6		0.46		0.75		0.92	

Where, TB = tassel blasting, FLS = first leaf senescence and SLS = second leaf senescence

The comparison of hybrids with BLUE mean values for grain yield and standard checks under CDHS showed varied performance of hybrids in mean grain yield (Table 8). BLUE mean values for grain yield of hybrids in CDHS ranged from 565.5kgha^-1^ (HB64) to 5393.2kgha^-1^ (HB95). Forty seven percent (45 hybrids) of tested hybrids provided higher grain yield than the mean (2392.1kgha^-1^).

**Table 8:** BLUEs for grain yield of 100 genotypes (96 hybrids and 4 standard checks) tested under CDHS, MDTS and FIRR for two years (2020 and 2021).

Hybrid	BLUE_GY			Hybrid	BLUE_GY			Hybrid	BLUE_GY		
	CDHS	MDRTS	FIRR		CDHS	MDRTS	FIRR		CDHS	MDRTS	FIRR
HB1	1554.1	1655.0	4808.8	HB35	3447.5	1385.96	4327.6	HB69	3152.0	2546.5	4814.1
HB2	1812.1	1677.1	4309.1	HB36	736.1	969.7	2919.7	HB70	3655.0	1069.9	3604.9
HB3	2140.8	1772.1	4603.0	HB37	2893.6	1691.2	4168.7	HB71	2055.0	1234.6	5162.9
HB4	2732.0	1852.5	4847.5	HB38	1928.9	1283.6	4391.6	HB72	2753.0	1636.1	4809.4
HB5	1792.1	1757.5	5201.8	HB39	3946.9	1439.9	3096.1	HB73	949.1	769.6	2005.8
HB6	2062.1	1088.5	4112.7	HB40	1411.7	839.4	4538.6	HB74	3027.3	1574.7	3348.2
HB7	1951.0	1600.0	3824.8	HB41	4771.8	1969.0	5542.0	HB75	2277.9	1522.3	4129.8
HB8	1983.5	440.6	5219.6	HB42	4010.6	1244.0	4714.0	HB76	689.5	718.9	3204.0
HB9	3754.7	1522.6	3894.4	HB43	5023.7	1767.8	4573.6	HB77	795.2	417.5	2481.6
HB10	1662.6	1207.8	3730.4	HB44	3253.5	1668.8	4291.8	HB78	2037.9	1322.7	4500.0
HB11	2161.8	1047.2	3361.6	HB45	1805.8	1072.6	4746.4	HB79	1443.7	1329.6	3742.1
HB12	2594.0	1486.6	2964.3	HB46	2904.8	1313.8	4479.4	HB80	1011.0	928.0	3321.9
HB13	1242.3	887.8	3409.0	HB47	4129.1	2313.9	4582.1	HB81	790.3	703.1	3469.0
HB14	676.5	807.8	2763.4	HB48	613.3	886.9	3688.4	HB82	2492.8	1471.0	4115.7
HB15	1285.9	426.5	3370.2	HB49	2904.4	998.8	3609.7	HB83	2824.1	1065.6	3100.4
HB16	1763.2	836.6	3199.3	HB50	1909.1	1451.0	5776.9	HB84	2513.5	1017.5	3280.5
HB17	2668.2	1183.0	5266.0	HB51	3011.1	1855.7	5396.7	HB85	4939.1	1499.5	4084.6
HB18	4218.0	1971.7	4880.5	HB52	1693.2	1488.3	4153.4	HB86	3443.7	1369.4	2955.0
HB19	2259.5	664.1	2712.5	HB53	2769.6	1028.3	4064.7	HB87	3578.8	1455.6	3574.7
HB20	840.8	545.5	1725.4	HB54	3729.4	679.7	3815.4	HB88	2872.1	813.3	3511.4
HB21	2373.7	1802.0	3505.9	HB55	2325.8	2113.3	5360.9	HB89	970.0	1166.2	3807.6
HB22	1882.1	1709.0	4023.1	HB56	3480.7	1587.5	4605.0	HB90	2775.7	872.2	4473.1
HB23	1443.7	831.6	2823.1	HB57	2832.3	1844.4	3706.7	HB91	4538.3	1960.5	4534.5
HB24	1066.2	706.9	2401.4	HB58	2816.6	1483.2	5390.8	HB92	1490.6	1110.6	3679.5
HB25	1931.2	1063.5	4353.8	HB59	1268.2	619.0	3464.1	HB93	1505.8	859.3	4137.5
HB26	1967.0	1290.6	4027.6	HB60	795.9	788.6	2263.7	HB94	2283.4	2274.2	4623.4
HB27	1352.6	895.6	2773.6	HB61	2703.6	1348.7	4613.0	HB95	5393.2	1676.9	5490.4
HB28	1607.9	555.7	3442.5	HB62	3559.1	1856.0	4767.6	HB96	2729.7	1096.3	3476.8
HB29	2527.3	1171.1	5845.5	HB63	1235.5	280.1	1950.7	HB97	2748.9	1386.5	6111.8
HB30	2822.0	935.6	4995.5	HB64	565.2	452.2	2199.4	HB98	2760.8	1657.8	5840.5
HB31	1579.4	661.2	2615.9	HB65	4292.6	1851.1	4178.0	HB99	3971.2	2434.2	4867.3
HB32	875.7	524.3	3002.5	HB66	3579.9	1077.1	2194.4	HB100	3117.1	1911.9	4643.6
HB33	2517.8	2155.9	5125.6	HB67	3191.9	1355.4	3598.4	Mean	2392.1	1288.9	3991.4
HB34	1453.9	1213.6	4066.0	HB68	2653.9	2063.1	4257.6	LSD	1210.5	772.8	1172.8

Nine hybrids showed better grain yield performance than all standard checks (heat tolerant check BH97, heat susceptible check BH98, commercial hybrids BH100 and BH99). Thirty five better yielding hybrids displayed 0.15% (HB72, 2753 kgha-1) to 96.2% (HB95, 5393.2kgha^-1^) yield advantage over the heat tolerant check BH97 (2748.9 kgha^-1^) and had desirable secondary features such as lower ASI, TB, good PASP and EASP. Nine (HB18, HB41, HB42, HB43, HB47, HB65, HB85, HB91 and HB95) of the highest-yielding hybrids showed yield advantages of between 0.99% (HB39) to 35.8% (HB95) compared to the best commercial check BH99. Combined drought and heat stress caused 66.9% yield reduction compared to FIRR.

Stress indices were determined using BLUE values to identify tolerant and susceptible genotypes under CDHS (S7 Table). Geometric mean productivity (GMP) values expressed 50% (48 hybrids) valued more than its mean (3063). The number of hybrids in the CDHS with stress indices that were larger or smaller than the mean ranged from 39 for SYI to 49 for MP identified their less sensitivity to CDHS. Six to seventeen hybrids recorded higher GMP, STI and MP values than standard checks (HB97, HB98, HB99 and HB100) depending on the check compared. Tolerance index (TOL) indicated that 40 hybrids had smaller values than the mean and twenty eight hybrids displayed lower TOL values over all checks. Forty three hybrids valued smaller SSI and higher STI indices compared to the mean. SSI index also indicated that nineteen to sixty two hybrids were CDHS tolerant compared with standard checks. Yield index discriminated that 9.4 to 50 percent of hybrids were less sensitive to CDHS comparing with the mean and checks. Yield stability index (YSI) identified 22 to 62 hybrids were tolerant to CDHS comparing with mean and checks. All estimated stress tolerance indices confirmed that hybrid HB18, HB41, HB43, HB47, HB85, HB91 and HB95 were more resistant to CDHS values than all standard checks.

BLUE of grain yield of hybrids under MDRTS varied from 208.1kgha-^1^ for BH63 to 2546.5kgha^-1^ for BH69. Forty eight percent of tested hybrids recorded higher grain yield compared to the mean grain yield (1288.9kgha-^1^) (Table 8). The number of better yielding hybrids over standard checks were varied from 1 to 38 depending on the check used. Average grain yield of hybrids under FIRR ranged from 1725.4kgha^-1^ (HB20) to 6111.8kgha^-1^(HB97). Fifty (52.1%) hybrids of the tested hybrids were superior in grain yield compared with the mean. Of these hybrids were those hybrids that showed better yield than the mean under MDRTS.

Twenty one hybrids recorded higher grain yield than commercial check HB100. Commercial check HB99 was out yielded by fourteen hybrids and heat susceptible check HB98 was out yielded by HB29 only. No hybrid out yielded best yielding heat tolerance check HB97 (6111.8kgha^-1^) under FIRR (Table 8). Generally drought stress caused various degree of yield reduction for all hybrids compared with full irrigation.

The number of hybrids under MDRTS with above-average or below-average stress indices varied from 42 for YSI to 54 for TOI (S6 Table). The most effective hybrids to test HB97 for drought stress included 14 hybrids for GMP, 28 hybrids for HM, 14 hybrids for STI, 1 hybrid for MP, 43 hybrids for YI, 77 hybrids for YSI, and most hybrids for TOI and SSI. According to drought stress indices, 7 hybrids for GMP, 1 hybrid for MP, 13 hybrids for HM, 7 hybrids for STI, 24 hybrids for YI, 56 hybrids for YSI and the majority of hybrids for TOI and SSI were more drought-tolerant than HB98. HB69 for all estimated indices, HB41 and HB55 for MP, HB47 and HB21 for SSI and HB70 exceeded commercial hybrid HB99 in drought tolerance. The best hybrids to HB100 for drought tolerant were twelve hybrids for GMP, fifteen hybrids for MP, ten hybrids for HM and STI, seventeen hybrids for SSI and YSI, nine hybrids for YI and 25% of tested hybrids for TOL. Nine hybrids HB18, HB41, HB47, HB51, HB55, HB69, HB91, HB94 and HB95 were found to be highly productive and drought tolerant by the majority of drought stress indices.

### Phenotypic and genetic correlations among traits measured under CDHS, MDRTS, and FIRR

**Fig 1:**
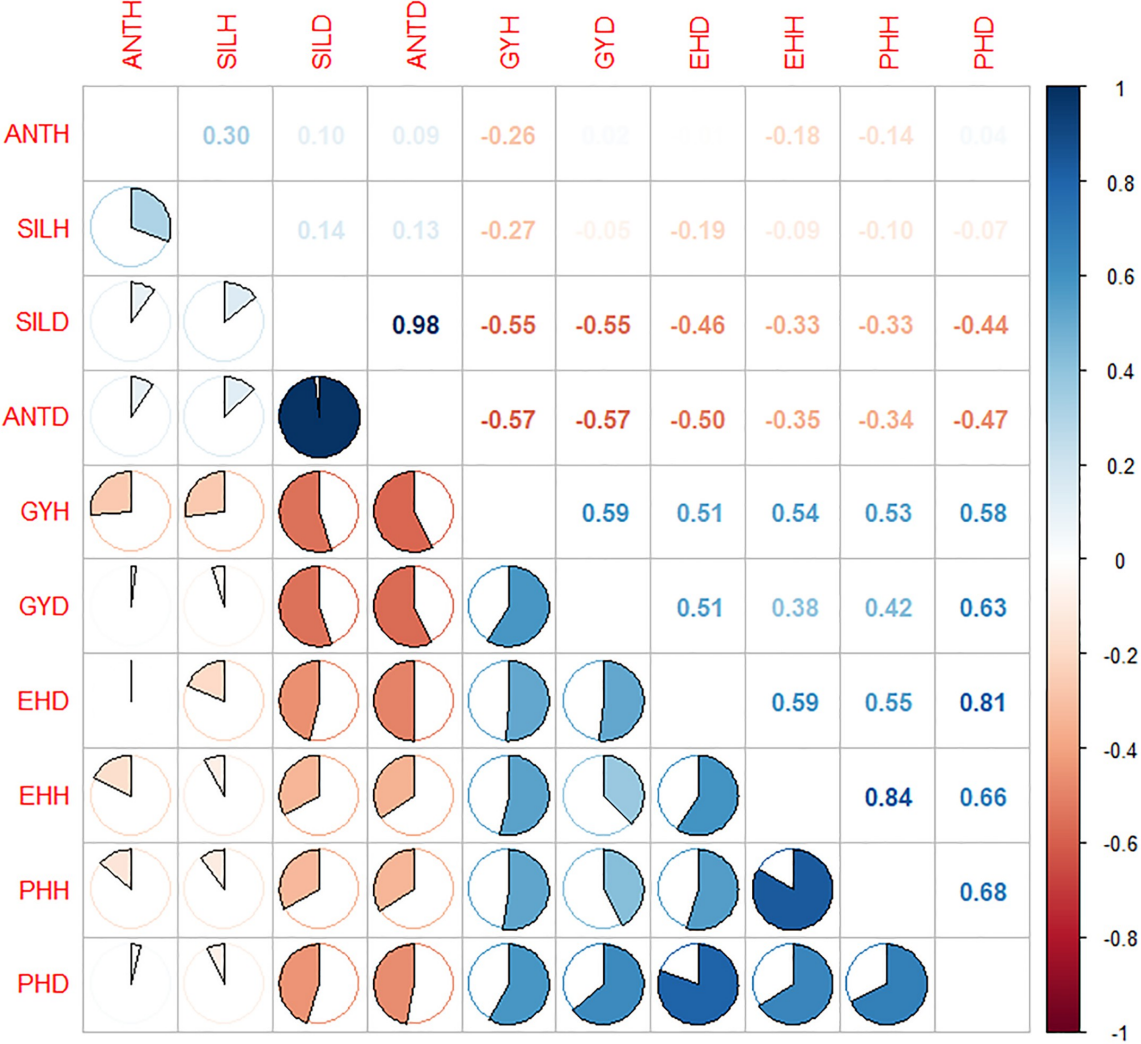
Phenotypic correlation among yield and yield components under CDHS with MDTS. Where, GYH, ANTH, SILH, PHH, EHH, HCH, PASPH, EASP = BLUE of Yield, Date to 50% anthesis date, Date to 50% silking date, Plant height, Ear height, husk cover, Plant aspect and Ear aspect under CHDS respectively while GYD, ANTD, SILD, PHD, EHD and HCD = BLUE of Yield, Date to 50% anthesis date, Date to 50% silking date, Plant height, Ear height, husk cover under MDTS accordingly.

Most of the agronomic traits recoded under CDHS and MDRTS exhibited significant phenotypic (Fig 1) and genotypic (Table 9) correlation with each other. Phenotypic correlation coefficients among agronomic characters under CDHS and MDRTS ranged between -0.75 to 0.98 whereas genotypic correlation coefficients of these traits varied from -1.00 to 1.00. Grain yield under MDRTS had very highly significant strong positive phenotypic correlation (r = 0.59) with grain yield under CDHS. Plant height and ear height under MDRTS and CDHS showed positive significant moderate phenotypic correlation with grain yield under CDHS while PASPH, EASPH, ANTD and SILD expressed significant negative phenotypic correlation with GYH. Phenotypic correlation coefficients for PHD with EHD and GYD were highly significant. ANTD and SILD recorded very highly significant moderate negative correlation with GYD

**Table 9:** Genotypic correlation among BLUE of yield and yield components under CHDS with MDRTS.

Traits	GYD	GYH	ANTD	ANTH	SILD	SILH	PHD	PHH	EHD
GYH	1.00***								
ANTD	-0.62***	-1.00***							
ANTH	-0.62***	-1.00***	0.89***						
SILD	-0.63***	-0.97***	1.00***	0.90***					
SILH	-0.59**	-1.00***	0.94***	0.93***	0.96***				
PHD	0.83***	1.00***	-0.56**	-0.54***	-0.57**	-0.47***			
PHH	0.79***	0.74***	-0.44**	-0.40***	-0.44**	-0.43**	1.00***		
EHD	0.74***	1.00***	-0.90**	-0.88***	-0.91***	-0.82***	0.97***	1.00***	
EHH	0.61***	0.73***	-0.46**	-0.45**	-0.45**	-0.48**	1.00***	0.92***	1.00***

Genotypic correlation coefficient between grain yield in the CDHS and MDRTS conditions showed very strong and highly significant positive relationship (Table 9). Grain yield also displayed very strong and highly significant positive genotypic correlation with PH and EH at both stress environments. In contest it had very strong and highly significant negative genotypic correlation with ANT, SIL and HC under CDHS and MDRTS. Days to 50% anthesis at CDHS and MDRTS were associated positively and significantly with SIL, HC, PASP and EASP whereas the association was negative and significant for PH and EH under CDHS and MDRTS. Other yield components also recorded highly significant positive or negative association with each other under CDHS and MDRTS.

**Fig 2:**
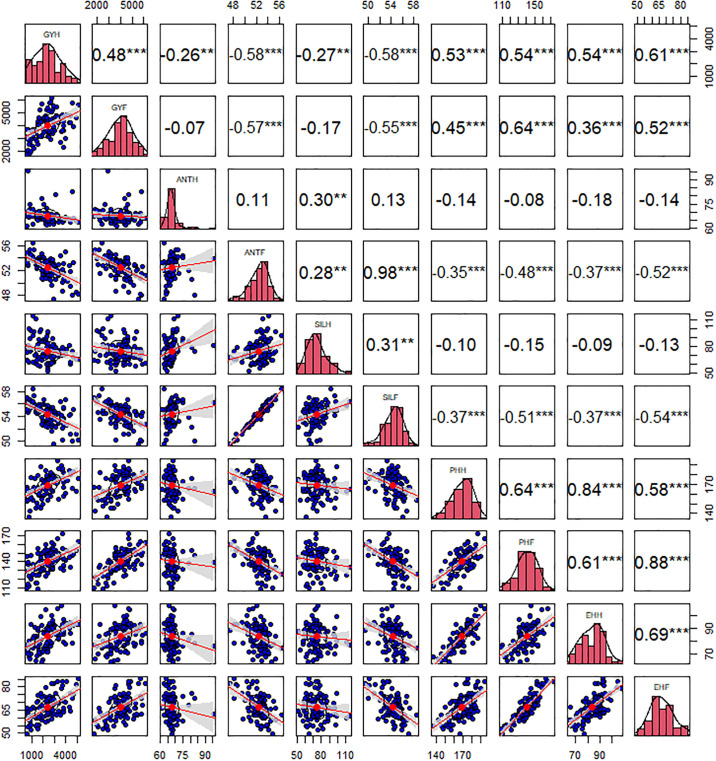
Phenotypic correlation among yield and yield components under CDHS with FIRR. Where, GYF, ANTF, SILF, PHF and EHF = BLUE of Yield, Date to 50% anthesis date, Date to 50% silking date, Plant height, Ear height, husk cover under FIRR accordingly. GYH and GHF expressed positive, strong and significant genotypic correlation (r = 0.84***) with one another and PHH, PHF, EHH and EHF whereas negative, strong and significant genotypic correlation with ANTH, ANTF, SILF, HCH, PASPH and EASPH. Genetic correlations for yield components were from strong to moderate significant positive or negative to each other and yield at CDHS and FIRR.

The phenotypic and genotypic correlation coefficients were determined among yield and yield components under CDHS and FIRR to discover the nature of the genotypic (Table 10) and phenotypic (Fig 2) association. Genotypic and phenotypic association among characters varied from -1 to 1.00 and -0.75 to 0.98, respectively. Grain yield under CDHS and FIRR exhibited moderate positive significant phenotypic relationship. Phenotypic association analysis displayed that yield related traits showed from strong to weak positive or negative significant correlation among themselves and yield under CDHS and FIRR.

**Table 10:** Genotypic correlation BLUE of yield and yield components under CDHS and FIRR.

Traits	GYF	GYH	ANTF	ANTH	SILF	SILH	PHF	PHH	EHF
GYH	0.84***								
ANTF	-0.61***	-0.84***							
ANTH	-0.38*	-1.00***	0.82***						
SILF	-0.60***	-0.81***	1.00***	0.82***					
SILH	-0.32ns	-1.00***	0.83***	0.93***	0.83***				
PHF	0.66***	1.00***	-0.67***	-0.42**	-0.67***	-0.37**			
PHH	0.72***	0.74***	-0.39*	-0.40**	-0.38**	-0.43**	1.00***		
EHF	0.55**	1.00***	-0.77***	-0.53**	-0.75***	-0.52**	0.97***	0.95***	
EHH	0.60***	0.73***	-0.45**	-0.45**	-0.44**	-0.48**	1.00***	0.92***	1.00***

**Fig 3:**
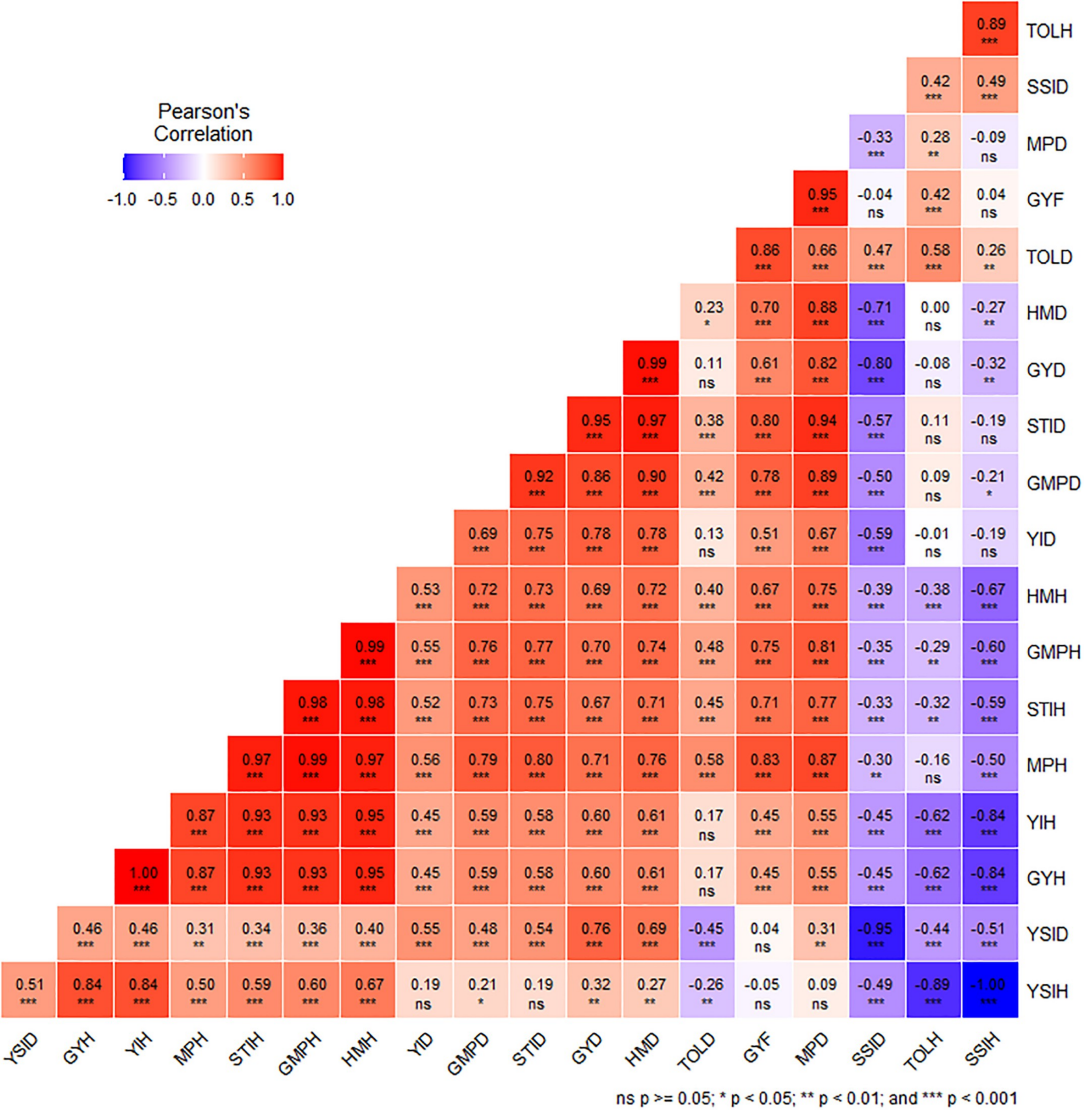
Phenotypic correlation of GYH, GYD and GYF with stess toleance indices unde CDHS and MDRTS. Where GYD = Grain yield under MDRTS, GYF = Grain yield under FIRR, GYH = Grain yield under CDHS, GMPH, MPH, HMH, SSIH, TOLH, STIH, YSIH and YIH are stress indices under CDHS, GMPD, MPD, HMD, SSID, TOLD, STID, YSID and YID are stress indices under MDRTS.

Correlation coefficients among GYH, GYD, GYF and stress tolerance indices were determined to identify the most tolerable stress tolerant criterion (Fig 3). GYH GYD and GYF presented a strong positive and significant association with all stress tolerance indices except SSID, SSIH and TOLH correlated strongly and negatively with GHY. Stress tolerance indices YIH, GMPH, MPH, HMH and STIH displayed highest positive correlation with GYH. Similarly, stress tolerance indices GMPD, HMD, STID, MPD and YID showed maximum positive correlation with GYD. Most stress tolerance indices under MDRTS had strong positive association with most of the stress tolerance indices under CDHS. But SSID, SSIH and TOLH exhibited weak positive or strong negative relationship with most stress tolerance indices except TOLH with SSIH which scored strong positive correlation (r = 0.89).

## Discussion

Tested hybrids derived from 24 inbred lines were selected from highly diversified drought tolerant, *Striga* resistant, heat tolerant and heat sensitive 271 inbred lines. These hybrids revealed huge genetic diversity for most importance agronomic traits under MDRTS, CDHS and FIR enabling the selection of CDHS resilient hybrids with broad adaptations. CDHS tolerant findings [13, 30–32] assured the prevalence of significant genetic variability for CDHS tolerance. Grain yield of hybrids under CDHS and FIRR was influenced by year variation indicating the yielding ability of hybrids varied from year to year. Environmental differences had huge influence on grain yield performance of maize genotypes to respond differently across environments [33, 34].

BLUE analysis was executed for grain yield to select best performing hybrids under stress and optimum conditions. Silveira et al. [35] selected superior maize genotypes using BLUE values. Thirty five hybrids demonstrated a 0.15% (HB72, 2753kgha^-1^) to 96.2% (HB95, 5393.2kgha^-1^) yield advantage over heat tolerant check BH97 (2748.9 kgha^-1^) and nine hybrids were better yielding than all standard checks under CDHS. Chiuta & Mutengwa, [32] identified high yielding four single crosses under CDHS. In this study, grain yield performance of forty eight percent of the evaluated hybrids exceeded the mean grain yield (1288.9kgha-^1^) and the number of best performing hybrids varied over each check and hybrid HB69 out yielded all standard checks under MDRTS. Chiuta & Mutengwa and Nelimor et al. [13, 32] identified maize genotypes which had better performance in grain yield compared with drought tolerant check. Thirty-three percent of tested single crosses showed better grain yield than mean under both CDHS and MDRTS, suggesting the presence of resistance genes to counter the CDHS. Most of the hybrids that showed higher yields compared to respective standard checks under MDRTS were those that displayed superior grain yield under CDHS demonstrating the possibility of developing combined heat and drought tolerance hybrids. Khatibi et al. [34] studied tolerance of maize hybrids to drought stress and found two maize hybrids less sensitive to water stress. Nelimor et al. and Nasser et al. [10, 13] identified maize genotypes which showed better yield performance than checks under heat stress, MDRTS and CDHS condition. They added that these genotypes showed 4% to 42% yield advantage at CDHS condition. Inbred lines exhibited better tolerant to CDHS as well as resistance to cope with individual stresses [31]. Cairns et al. [12] presented ten testcrosses coping drought and combined heat and drought stress.

The mean grain yield of hybrids at FIRR varied from 1725 kgha^-1^ (HB20) to 6111 kgha^-1^ (HB97). Fifty hybrids were superior in grain yield to the average. One to twenty one hybrids yielded higher than three standard checks. But there was no hybrid that out yielded the best performing heat tolerant check HB97.

Different stress indicators have been determined to select tolerant and sensitive genotypes based on their performance in stressed and non-stressed environments [34, 36]. Stress-tolerant genotypes exhibit highest values for tolerance indices MP, HM, GMP, STI, YI, and YSI and lowest values for SSI and TOL [34, 36]. Majority of stress indices indicated that hybrids HB18, HB41, HB42, HB43, HB85, HB91 and HB95 were tolerant to CDHS and had better stress tolerance indices compared to standard checks. The smallest indices for MP, HM, GMP, STI, YI, and YSI, as well as greater values for SSI and TOI were found in hybrid HB64 and HB20 which were identified as being very sensitive to CDHS. Twelve hybrids (HB4, HB5, HB18, HB33, HB41, HB47, HB51, HB55, HB69, HB91, HB94, and HB95) were identified as being high yielders and drought tolerant by the majority of drought stress indices. Khatibi et al. [34] found that two maize hybrids (SC647 and KSC70) could withstand water stress by calculating drought tolerance indices. Most stress indices revealed that hybrids HB18, HB41, HB91, and HB95 were high yielders and resilient to individual and combined stresses. Scholars [13, 34, 37] identified resistance to drought, heat and CDHS resistant genotypes using stress tolerance indices.

Designing appropriate breeding strategies for development of CDHS resilient maize hybrids needs understanding and analysis of combining ability under drought and CDHS environments. Partitioning genotypic mean squares of hybrids revealed that variances due to female (set) and male (set) were very highly significant (p<0.001) for most traits under all testing environments demonstrating the presence of sufficient genetic variations among the parental lines and their hybrids. Former studies [10, 24, 38] reported the presence of genetic differences among maize genotypes under drought and CDHS. Under all research environments, the variance of female (set) by male (set) was highly significant for grain yield, and under MDRTS, it was highly significant for days to anthesis and days to silking.

GCA variance was larger compared to SCA variance for most studied traits revealing additive gene action was more important than non-additive gene action. Scholars demonstrated the predominance of additive gene action over the non-additive gene action under managed drought stress, CHDS and well watered environments [10, 38]. Akaogu et al [39] and Badu-Apraku et al [40] explained the importance of additive gene action over non-additive gene action under managed drought stress and well watered conditions. All traits controlled by preponderance of additive gene action under CDHS except grain yield, which was governed by non-additive gene action [32]. While grain yield was controlled by preponderance of non-additive gene action under CDHS [41]. Umar et al. [42] and Oyekunle et al. [23] observed that most traits were controlled by non-additive gene action under drought and optimum growing conditions. For grain yield under all environmental conditions, year by female (set) interaction variance was significant, showing the response of parents as female for this trait varied with year. Variance for year x male (set) interaction was highly significant for grain yield under CHDS and FIRR. The decomposition of hybrids variance in female and male variance showed the existence of significant environment by female and environment by male variances [38, 41] for yield. Variance for year x female x male (set) interaction was highly significant for anthesis silking interval under CDHS and significant for plant aspect under MDRTS.

Under CDHS, six inbred lines displayed positively significant or non-significant positive GCA effects for grain yield and ear placement as well as significant or non-significant negative GCA effects for 50% days to anthesis and silking as they combined both as female and male parents. This implied that these lines could be potential for deriving CDHS tolerant high yielding and early maturing maize hybrids. Chiuta and Mutengwa [32] identified five inbred lines that were good general combiner for grain yield under CDHS. Significant grain yield GCA-female effects for parents showed that maternal effects played a great role in the inheritance of grain yield. Furthermore, the results specified that alleles which favor these traits can be easily introduced into tropical maize to improve the yielding potential and maturity of hybrids. Significant high GCA values have shown the presence of additive genetic actions which is fixable and transfers parental characters in to their hybrids [24, 43]. Among lines with positively GCA effects for grain yield, three (Entry 7, 9 and 10) were tolerant and the remaining five (Entry 14, 18, 19, 21 and 22) were susceptible to heat. Researching to get CDHS tolerant lines and hybrids by Chiuta & Mutengwa [32] identified five good general combiner lines for grain yield, of which three were tolerant and two were susceptible to drought stress at seedling stage. Comparatively, under the MDRTS, seven lines (Entry 7, 9, 10, 18, 19, 21 and 22) manifested positive significant or non-significant GCA male and female effects. These results suggested that these parents may be significant donors of valuable alleles for grain yield when they are used as both male and female parents. Parents 19 and 22 both demonstrated favorable GCA effects indicating they would contribute good genes for the creation of early maturing hybrids. High significant GCA effects for grain yield and short days to 50% tasseling and days to 50% silking are important for enhancing yielding potential and early maturity [42]. Malook et al. and Issa et al. [44, 45] identified inbred lines which had highest and positively significant GCA effects for grain yield and other agronomic traits under drought stress. GCA-female and male effects favorably influenced grain yield under FIRR, revealing the ability of lines eight inbred lines (Entry 4, 5, 6, 9, 10, 15, 19 and 22) to develop high yielding hybrids and good parental source of genes for the trait, in contrast to inbred lines 11 and 12, which showed unfavorable GCA-female effects for the same trait. Chiuta & Mutengwa and Abd-elnaser & El-latif [32, 47] stated that the presence of good or poor general combiner inbred lines for grain yield under FIRR. Off them, four lines (Entry 6, 15, 19 and 22) showed significant negative GCA female effects for plant height, ear placement, anthesis and silking days. Lines desirable for development of long or short stature hybrids were recommended by Abd-elnaser & El-latif [47]. In general, four parental lines 9, 10, 19 and 22 may be a source of useful genes for the production of high yielding hybrids under CDHS, MDRTS, and FIRR conditions. Four inbred lines which showed positive GCA-female and GCA-male effects for GY under CDHS and heat stress, were chosen by Osuman et al. [38].

Hybrids with significant or non-significant negative and positive SCA effects were recorded under the three research conditions. Eight cross combinations under CDHS, nine single crosses in MDRTS and four single crosses under FIRR were good specific combiners for grain yield. Of good specific combiner single crosses under CDHS, two single crosses 1 x 7 and 10 x 1 were crosses of heat tolerant parents and the other two single crosses 5 x 19 and 7 x 20 were combinations from heat tolerant and heat susceptible parental lines while the remaining four (17 x 14, 19 x 16, 19 x 15 and 13 x 21) were generated from heat susceptible parental lines. Tolerant-tolerant combination of 10 x 1, tolerant-susceptible combinations of 5 x 17, 7 x 20, 23 x10, 23 x 12, 24 x 10, and susceptible-susceptible combinations of 13 x 22, 13 x 24 and 18 x 16 were well specific combiner for grain yield under MDRTS. Single cross 10 x1 and 7 x20 were good specific combiner for grain yield under both MDRTS and CDHS. Chiuta & Mutengwa [32] found four hybrids that were well specific combiner under CDHS for grain yield. They added that three of these hybrids were the combinations of drought tolerant and drought susceptible lines and the remaining one hybrid obtained from crossing two drought susceptible lines at seedling stage. Two of five best performing hybrids in grain yield under CDHS, were obtained from crossing of drought tolerant and susceptible lines [10] and the rest three resulted from crossing of drought tolerant lines. In contrast, Badu-Apraku et al. [46] suggested that stress tolerant hybrids could be obtained from the combination of stress tolerant inbred lines. Selected hybrids under CDHS provided high yield coupled with small tassel blast and stress tolerance indicator indices indicating their good specific combining ability and resilient to CDHS though they had high positive non-significant SCA effects.

Grain yield under CDHS revealed highly significant strong (r = 0.59***) and moderate (r = 0.48***) phenotypic correlation coefficient with grain yield for MDRTS and FIRR accordingly suggesting the existence common genetic mechanisms controlling yield under CDHS and MDRTS. Similar findings were stated by many researchers [12–14, 45]. Tandzi et al. [31] found positive and moderate correlation between grain yield under heat and CDHS. Contrastingly, Cairns et al. and Nelimor et al. [12, 13] found weak phenotypic correlation between grain yield under stress and non-stress conditions. Genotypic correlation of grain yield under CDHS with grain yield under MDRTS and FIRR were strong, positive and highly significant (r = 1 for CDHS and MDRTS, r = 0.84 for CHDS and FIRR) indicating the presence of common genetic mechanisms governing yielding ability under these testing environments. GYH GYD and GYF were strongly, positively and significantly associated with all stress tolerance indices except SSID, SSIH and TOLH which correlated strongly and negatively with GHY. This implies that these indices and BLUEs of grain yield could be able to select tolerant single crosses under MDRTS and CDHS conditions. Tandzi et al. [31] reported the correlation of shoot weight of maize to all stress indices under drought, heat and combined stresses. Stress tolerance indices YIH, GMPH, MPH, HMH and STIH displayed highest positive correlation with GYH. Similarly, stress tolerance indices GMPD, HMD, STID, MPD and YID showed maximum positive correlation with GYD. Positive correlation of these indices with grain yield under heat stress agreed with the of results of Abd-Elnaser and Latif, Longmei et al. and Chand et al. [47–49]. Most stress tolerance indices under MDRTS had strong positive association with most of the stress tolerance indices under CDHS indicating their ability to identify multiple stress tolerance single crosses. Positive strong association of stress tolerance indices between two or more stresses designated the presence of tolerant genotypes under both stresses [31].

Tassel blasting is among the most important secondary trait which is very necessary for selection of maize genotypes under heat [50] and CDHS [14]. The tassel blasting score of most hybrids and parents were valued to zero with range of zero to 3.5 (HB76) for hybrids and 0 to 1.0 (23) for parents. Single cross HB76 resulted from combination of two heat susceptible parents and had low BLUE value for grain yield (689.5kgha^-1^). BH41, HB43 and HB47 were among single crosses with high BLUE value for grain yield and zero blast score. These single crosses developed from heat tolerant and heat susceptible parents scored zero tassel blast except parent 7 with blast value of 0.75. In contrast, other single crosses with high BLUE values and developed from the combination of two susceptible parents, were HB85, HB91 and HB95 with zero blast values. Parents of these hybrids also valued blast score of zero other than parent 15 with blast value of 0.75. Chiuta & Mutengwa [32] obtained high yielding hybrids from the combination of drought tolerant and drought susceptible lines and from crossing two drought susceptible lines. Hybrids with low tassel blasting score provided high grain yield where as those with high tassel blast score produced low yield [14, 50]. Developing drought, heat and CDHS tolerance maize increased maize yield under current and future climates [51]. They added the advantage of combined heat and drought tolerant maize is more than twice that of heat or drought tolerance and increased with temperature increment. Identified drought, heat and CDHS tolerant maize genotypes can be used as source genetic materials for development other biotic and abiotic stress tolerant lines and hybrids.

## Conclusion

Hybrids showed highly significant differences for grain yield and other agronomic traits under FIRR, MDRTS and CDHS indicating the presence of sufficient genetic variability for selection of MDRTS and CDHS tolerant genotypes. The majority of hybrids that showed improved grain yield over their respective standard checks under MDRTS presented improved grain yield over the same checks under CDHS. BLUE values revealed the existence of hybrids that had better performance under both MDRTS and CDHS conditions. Most stress indices discovered hybrids HB18, HB41, HB91 and HB95 were high yielding and resilient to MDRTS and CDHS. These hybrids were well specific combiner for grain yield under CDHS. Hybrids HB41, HB91 and HB95 were also selected due to their and their parents’ low tassel blast values. Significant and positive correlation of grain yield under MDRTS and CDHS signified the existence of common genetic mechanisms controlling yield under MDRTS and CDHS. Stress tolerance indices YI, GMP, MP, HM and STI were identified as best selecting indices under both stresses. Four parental lines 7, 9, 10 and 19 were well general combiner for grain yield and early maturity under MDRTS and CDHS and could be very useful gene source for hybridization. For most traits, GCA variances were larger than SCA variances in each testing condition indicating the existence of common genetic mechanism for controlling these traits across testing conditions. Promising hybrids should be evaluated further under different drought and CDHS conditions for commercialization. The study’s findings concluded that the development of CDHS tolerance in maize genotype can mitigate anticipated yield losses and stabilize maize productivity in CDHS vulnerable areas. Development of maize genotypes with CDHS tolerance is a sound strategy to enhance adaptation to climate change in Africa and worldwide.

## Supporting information

S1 Table Code for twelve tassel blast tolerant and twelve tassel blast susceptible lines and their cross pattern. (DOCX)

S2 TableEquations and references of stress tolerance indices used to assess maize hybrids.Where YP, $\bar{\text{YP}}$ grain yield and mean grain yield respectively under optimum condition and YS, YS grain yield and mean grain yield respectively under stress condition.(DOCX)

S3 Table Mean squares from combined analyses for grain yield and other traits of 96 hybrids involving lines with varying resistance to tassel blast tested under combined drought and heat stress, managed drought stress and full irrigation for two years (2020 and 2021). (DOCX)

S4 Table SCA effect estimates for yield and yield related traits in 96 crosses produced through factorial mating of 24 inbred lines under combined heat and drought stress. (DOCX)

S5 Table SCA effect estimates for yield and selected yield related traits in 96 single crosses produced through factorial mating of 24 inbred lines under managed drought stress. (DOCX)

S6 Table SCA effect estimates for grain yield and selected yield related traits in 96 single crosses produced through factorial mating of 24 inbred lines under full irrigation. (DOCX)

S7 Table Stress tolerance indices of genotypes for drought and combined heat and drought stresses. (DOCX)

## Abbreviations

BLUE: Best linear unbiased estimate
CDHS: Combined drought and heat stress
FAO: Food and agricultural organization
FIRR: Full irrigation
GCA: General combining ability
GMP: Geometric mean productivity
HB: Hybrid
HM: Harmonic mean
IITA: International Institute of tropical Agriculture
MDRTS: Managed drought stress
MP: Mean productivity
SCA: Specific combining ability
SSA: Sub-Saharan Africa
SSI: Stress susceptibility index
STI: Stress tolerance index
TOI: Tolerance index
YI: Yield index
YSI: Yield stability index

## References

[1] Prasanna B. M., Cairns J. E., Yoseph P. H. Z., Dan B., and Manje M., “Beat the stress: breeding for climate resilience in maize for the tropical rainfed environments Climate Resilient Maize for Asia,” *Theor*. *Appl*. *Genet*., vol. 134, no. 6, pp. 1729–1752, 2021, 33594449 10.1007/s00122-021-03773-7 PMC7885763

[2] Poole N., Donovan J., and Erenstein O., “Viewpoint: Agri-nutrition research: Revisiting the contribution of maize and wheat to human nutrition and health,” *Food Policy*, vol. 100, p. 101976, 2021, doi: 10.1016/j.foodpol.2020.101976 32963420 PMC7499093

[3] FAO, “FAOstat Food and Agriculture organization of the united nations, Rome.,” 2021.

[4] Abate T., Shiferaw B., Menkir A., Wegary D., Kebede Y., and Tesfay K., “Factors that transformed maize productivity in Ethiopia,” *Food Sci*., no. July, 2015, doi: 10.1007/s12571-015-0488-z

[5] Tsedeke A., Monica F., Tahirou A., Girma K.T., Rodney L., Paswel M., et al., “Characteristics of maize cultivars in Africa: How modern are they and how many do smallholder farmers grow?,” *Agric*. *Food Secur*., vol. 6, no. 1, Mar. 2017, doi: 10.1186/S40066-017-0108-6 32983427 PMC7507799

[6] Ertiro T. B., Beyene Y., Das B., Mugo S., Olsen H.M., Roikeh S., et al., “Combining ability and testcross performance of drought-tolerant maize inbred lines under stress and non-stress environments in Kenya,” *Plant Breed*., vol. 205, pp. 197–205, 2017, doi: 10.1111/pbr.12464 28781399 PMC5518761

[7] Schussler J. R. and Westgate M. E., “Maize Kernel Set at Low Water Potential: I. Sensitivity to Reduced Assimilates during Early Kernel Growth,” 1991, doi: 10.2135/cropsci1991.0011183X003100050023x

[8] Adewale S. A., Akinwale R. O., Fakorede M. A. B., and Badu-apraku B., “Genetic analysis of drought-adaptive traits at seedling stage in early-maturing maize inbred lines and field performance under stress conditions,” *Euphytica*, vol. 0123456789, 2018, doi: 10.1007/s10681-018-2218-z

[9] M. Aslam, M. A. Maqbool, and R. Cengiz, *Drought Stress in Maize (Zea mays L*.*) Effects*, *Resistance Mechanisms*, *Global Achievements and Biological Strategies for Improvement*, no. November. 2015.

[10] L. M. Nasser, B. Badu-Apraku, V. E. Gracen, and H. N. A. Mafouasson, “Combining Ability of Early-Maturing Yellow Maize Inbreds under Combined Drought and Heat Stress,” *Agronomy*, no. November, pp. 1–26, 2020.

[11] Costa M. V. J. D. A., Ramegowda Y., Ramegowda V. R., Karaba N. N., Sreeman S. M., and Udayakumar M., “Combined Drought and Heat Stress in Rice: Responses, Phenotyping and Strategies to Improve Tolerance,” vol. 28, no. 3, 2021, doi: 10.1016/j.rsci.2021.04.003

[12] Cairns J., Crossa J., HZaidi P., Grudloyma P., Sanchez G., Araus L.J., et al., “Identification of Drought, Heat, and Combined Drought and Heat Tolerant Donors in Maize,” 2013, doi: 10.2135/cropsci2012.09.0545

[13] Nelimor C., Badu-Apraku B., Tetteh A. Y., and N’guetta A. S. P., “Assessment of Genetic Diversity for Drought, Heat and Combined Drought and Heat Stress Tolerance in Early Maturing Maize Landraces,” *Plants*, pp. 1–19, 2019. 31744251 10.3390/plants8110518 PMC6918211

[14] Meseka S., Menkir A., Bossey B., and Mengesha W., “Performance Assessment of Drought Tolerant Maize Hybrids under Combined Drought and Heat Stress,” *agronomy*, vol. 8, no. 274, pp. 1–17, 2018, doi: 10.3390/agronomy8120274 33304638 PMC7672364

[15] Campos H., Cooper M., Habben J. E., Edmeades G. O., and Schussler J. R., “Improving drought tolerance in maize: a view from industry,” vol. 90, pp. 19–34, 2004, doi: 10.1016/j.fcr.2004.07.003

[16] Schmidt J., Tricker P. J., Eckermann P., Kalambettu P., Garcia M., and Fleury D., “Novel Alleles for Combined Drought and Heat Stress Tolerance in Wheat,” vol. 10, no. January, pp. 1–14, 2020, 10.3389/fpls.2019.01800 PMC7005056 32082351

[17] Beyene Y., Mugo S., Semagn K., Asea G., and Trevisan W., “Genetic distance among doubled haploid maize lines and their testcross performance under drought stress and non-stress conditions,” *Euphytica*, pp. 379–392, 2013, doi: 10.1007/s10681-013-0867-5

[18] Menkir A., Crossa J., Meseka S., and Bossey B., “Stacking Tolerance to Drought and Resistance to a Parasitic Weed in Tropical Hybrid Maize for Enhancing Resilience to Stress Combinations,” *Front*. *Plant Sci*., vol. 11, no. February, pp. 1–16, 2020, doi: 10.3389/fpls.2020.00166 32194590 PMC7061855

[19] Akaogu I. C., Badu-Apraku B., Tongoona P., Ceballos H., Gracen V., Offei S. K., et al., “Inheritance of Striga hermonthica adaptive traits in an early-maturing white maize inbred line containing resistance genes from Zea diploperennis,” *Plant Breed*., vol. 138, no. 5, pp. 546–552, 2019, doi: 10.1111/pbr.12707

[20] Kumar B., Choudhary M., Kumar P., Kumar1 K, Kumar1 S, Singh B. K, et al., “Population Structure Analysis and Association Mapping for Turcicum Leaf Blight Resistance in Tropical Maize Using,” *Genes (Basel)*., vol. 13, no. 618, pp. 1–14, 2022. 10.3390/genes13040618 PMC9030036 35456424

[21] Velez-Torres M., Garcıa-Zavala J. J., Hernandez-Rodrıguez M., Lobato-Ortiz R., Lopez-Reynoso J., Benıtez-Riquelme I., et al., “Genomic prediction of the general combining ability of maize lines (*Zea mays* L.) and the performance of their single crosses,” *Plant Breed*., no. March, pp. 1–9, 2018, doi: 10.1111/pbr.12597

[22] Elmyhun M., Liyew C., Shita A., and Andualem M., “Combining ability performance and heterotic grouping of maize (*Zea mays*) inbred lines in testcross formation in Western Amhara, North West Ethiopia,” *Cogent Food Agric*., vol. 6, no. 1, 2020, doi: 10.1080/23311932.2020.1727625

[23] Oyekunle M., Badu-Apraku B., Hearne S., and Franco J., “Genetic diversity of tropical early-maturing maize inbreds and their performance in hybrid combinations under drought and optimum growing conditions,” vol. 19, no. 4, pp. 332–341, 2015.

[24] Annor B., Badu-apraku B., Nyadanu D., Akromah R., and Fakorede M. A. B., “Testcross performance and combining ability of early maturing maize inbreds under multiple-stress environments,” *Sci*. *Rep*., no. September, pp. 1–11, 2019, doi: 10.1038/s41598-019-50345-3 31551523 PMC6760492

[25] Mittler R., “Abiotic stress, the field environment and stress combination,” *TRENDS Plant Sci*. *Vol*.*11*, vol. 11, no. 1, 2006, doi: 10.1016/j.tplants.2005.11.002 16359910

[26] Gabasawa A., Mohammed H., and Yusuf A., “Biological Nitrogen Fixation and Pod Yield of Groundnut (Arachis hypogaea L.) as Influenced by a Salt-affected Alfisol at Kadawa, Nigeria,” *Int*. *J*. *Plant Soil Sci*., vol. 3, no. 11, pp. 1479–1489, 2014, doi: 10.9734/ijpss/2014/11391

[27] RCoreTeam, “R: A language and environment for statistical computing. R Foundation for Statistical Computing, Vienna, Austria.,” 2020. Accessed: Jan. 20, 2022. [Online]. https://www.eea.europa.eu/data-and-maps/indicators/oxygen-consuming-substances-in-rivers/r-development-core-team-2006.

[28] A. R. Hallauer, M. J. Carena, and Miranda Filho J.B., *Quantitative Genetics in Maize Breeding*. 2010.

[29] He T. and Li C., “Harness the power of genomic selection and the potential of germplasm in crop breeding for global food security in the era with rapid climate change,” *Crop Sci*., vol. 8, no. 5, pp. 688–700, 2020, doi: 10.1016/j.cj.2020.04.005

[30] Nelimor C., Badu-apraku B., and Tetteh A. Y., “Assessing the Potential of Extra-Early Maturing Landraces for Improving Tolerance to Drought, Heat, and Both Combined Stresses in Maize,” *Agronomy*, pp. 1–23, 2020.

[31] Tandzi L. N., Bradley G., and Mutengwa C., “Morphological Responses of Maize to Drought, Heat and Combined Stresses at Seedling Stage,” *J*. *Biol*. *Sci*., vol. 19, pp. 7–19, 2019, doi: 10.3923/jbs.2019.7.16

[32] Chiuta N. E. and Mutengwa C. S., “Combining Ability of Quality Protein Maize Inbred Lines for Yield and Morpho-Agronomic Traits under Optimum as Well as Combined Drought and,” *Agron*. *Artic*., vol. 10, no. 184, pp. 1–14, 2020.

[33] Al-Naggar A. M. M., Shafik M. M., and Musa R. Y. M., “AMMI and GGE biplot analyses for yield stability of nineteen maize genotypes under different nitrogen and irrigation levels,” *Plant Arch*., vol. 20, no. 2, pp. 4431–4443, 2020.

[34] Khatibi A., Omrani S., Omrani A., Shojaei S. H., Mousavi S. M. N., Illés Á., et al., “Response of Maize Hybrids in Drought-Stress Using Drought Tolerance Indices,” *Water*, vol. 14, no. 1012, pp. 1–10, 2022.

[35] Silveira E. S., Silva A. F., Santos B. N., Hugo G., and Oliveira F., “Morphological characterization and selection of maize genotypes for the semiarid region,” *Res*. *sqaure*, pp. 1–17, 2022, doi: 10.21203/rs.3.rs-1538344/v1 License:

[36] Aberkane H., Belkadi B., Kehel Z., Maltouf A. F., Meheesi S., Amri A., et al., “Assessment of Drought and Heat Tolerance of Durum Wheat Lines Derived from Interspecific Crosses Using Physiological Parameters and Stress Indices,” *Agronomy*, vol. 11, no. 695, pp. 1–20, 2021.

[37] Yahaya M. A., Shimelis H., Nebi B., Mashilo J., and Pop G., “Response of African Sorghum Genotypes for Drought Tolerance under Variable Environments,” *Agronomy*, vol. 13, no. 557, pp. 1–27, 2023.

[38] Osuman A. S., Badu-Apraku B., Ifie B.E., Nelimor C., Tongoona P., Obeng-Bio E., et al., “Combining Ability and Heterotic Patterns of Tropical Early-Maturing Maize Inbred Lines under Individual and Combined Heat and Drought Environments,” *Plants*, pp. 1–22, 2022. 35631790 10.3390/plants11101365 PMC9146004

[39] Badu-Apraku B., Yallou C. G., Obeng-Antwi K., Alidu H., Talabi A. O., Annor B., et al. “Yield Gains in Extra-Early Maize Cultivars of Three Breeding Eras under Multiple Environments,” *Agron*. *J*. *•*, vol. 109, no. 2, 2017, doi: 10.2134/agronj2016.10.0566

[40] Badu-Apraku B., Oyekunle M., Akinwale R. O., and Lum A. F., “Combining Ability of Early-maturing White Maize Inbreds under Stress and Nonstress Environments,” *Agron*. *jorunal*, vol. 103, no. 2, 2011, doi: 10.2134/agronj2010.0345

[41] O. A. Bhadmus, O.A. Badu-Apraku, Baffour. Adeyemo and A. L. Ogunkanmi, “Genetic Analysis of Early White Quality Protein Maize Inbreds and Derived Hybrids under Low-Nitrogen and Combined,” 2021. 10.3390/plants10122596 PMC8706249 34961067

[42] Umar U. U., Ado S. G., Aba D. A., and Bugaje S. M., “Estimates of Combining Ability and Gene Action in Maize (*Zea mays* L.) Under Water Stress and Non-stress Conditions,” *J*. *Biol*. *Agric*. *Healthc*., vol. 4, no. 25, pp. 247–254, 2014.

[43] Noëlle1 M. A. H, Vernon K. R., G, Martin Y. A, Laouali M. N, Liliane T. N, et al. “Combining Ability and Gene Action of Tropical Maize (*Zea mays* L.) Inbred Lines under Low and High Nitrogen Conditions,” *J*. *Agric*. *Sci*., vol. 9, no. 4, pp. 222–235, 2017, doi: 10.5539/jas.v9n4p222

[44] Malook S., Ali Q., Ahsan M., Shabaz M. K., and Waseem M., “Combining ability analysis for evaluation of maize hybrids under drought stress Combining ability analysis for evaluation of maize hybrids under drought stress,” *J*. *Natl*. *Sci*. *Found*. *Sri Lanka*, vol. 44, no. 2, 2016, doi: 10.4038/jnsfsr.v44i2.8003

[45] Issa Z. M. M., Nyadanu D., Richard A., Sangare A. R., Adejumobi I. I., and Ibrahim D., “Inheritance and combining ability study on drought tolerance and grain yield among early maturing inbred lines of maize (*Zea mays* L.),” *J*. *Plant Breed*. *Crop Sci*., vol. 10, no. June, pp. 115–127, 2018, doi: 10.5897/JPBCS2017.0703

[46] Badu-Apraku B. and Akinwale R. O., “Cultivar evaluation and trait analysis of tropical early maturing maize under Striga -infested and Striga -free environments,” *F*. *Crop*. *Res*., vol. 121, pp. 186–194, 2011, doi: 10.1016/j.fcr.2010.12.011

[47] Abd-Elnaser M. G. and El-Latif M. S. A., “Combining ability and heterosis for grain yield and its components in some maize inbred lines under drought stress,” 2022.

[48] Longmei N., Gill G. K., Kumar R., and Zaidi P. H., “Selection indices for identifying heat tolerant of maize (*Zea mays*),” *Indian J*. *Agric*. *Sci*., vol. 93, pp. 46–50, 2023, doi: 10.56093/ijas.v93i1.108617 Selection.

[49] H. Chand, P. Kayastha, B. K., B. Pandey, B. R. Magar, J. Bhandari, et al., “Evaluation of heat stress tolerance in wheat (Triticum aestivum L.) genotypes using stress tolerance indices in the western region of Nepal,” vol. 1878, no. 6, pp. 145–152, 2022.

[50] Noor J. J., Vinayan M. T., Umar S., Devi P., Iqbal M., and Seetharam K., “Morpho-physiological traits associated with heat stress tolerance in tropical maize (*Zea mays* L.) at reproductive stage,” *Aust*. *J*. *Crop Sci*., vol. 13, no. 04, pp. 536–545, 2019, doi: 10.21475/ajcs.19.13.04.p1448

[51] Tesfaye K., Gbegbelegbe S., Cairns J. E., Shiferaw B., Prasanna B. M., Sonder K., et al., “International Journal of Climate Change Strategies and Management Article information:,” *Int*. *J*. *Clim*. *Chang*. *Strateg*. *Manag*., vol. 7, no. 3, pp. 247–271, 2015.

