# Supplementary material for: Genetic analysis of tolerance to combined drought and heat stress in tropical maize
Source: PLoS One. 2024 Jun 20;19(6):e0302272. doi: 10.1371/journal.pone.0302272 (PMC11189248; doi:10.1371/journal.pone.0302272)
Supplement: S1 Table — (DOCX) [file pone.0302272.s001.docx]

Supplementary table 1.Code for twelve tassel blast tolerant and twelve tassel blast susceptible lines and their cross pattern

|  | | Female |  | Male | Code for their hybrids |
| --- | --- | --- | --- | --- | --- |
| Code | | Heat Stress tolerant S6 lines derived from bi-parental crosses of white DTSTR lines | Code | Heat Stress tolerant lines derived from DTSTR BCs containing temperate germplasm |  |
|  | | SET-1 |  | SET 2 | HB1-HB16 |
| 1 | | TZISTR1198/TZISTR1152-9-1-1-1-B-B | 5 | 1131(LH82-N1)-B/IITATZISTR1108-1-1-1-1-B |  |
| 2 | | TZISTR1198/TZISTR1152-19-1-1-1-B-B | 6 | 1131(LH82-N1)-B/IITATZISTR1108-4-1-1-1-B |  |
| 3 | | TZISTR1198/TZISTR1152-28-1-1-1-B-B | 7 | 1131(LH82-N1)-B/IITATZISTR1108-28-1-1-1-B |  |
| 4 | | TZISTR1199/TZISTR1152-130-1-1-1-B-B | 8 | 1133 LH59-N1-B/IITATZISTR1110-5-1-1-1-B |  |
|  | | Heat Stress tolerant lines derived from DTSTR BCs containing temperate germplasm |  | Heat Stress tolerant S6 lines derived from bi-parental crosses of white DTSTR lines |  |
|  | | SET-3 |  | SET1 | HB17-HB32 |
| 9 | | 1133 LH59-N1-B/IITATZISTR1110-60-1-1-1-B | 1 | TZISTR1198/TZISTR1152-9-1-1-1-B-B |  |
| 10 | | 1133 LH59-N1-B/IITATZISTR1110-62-1-1-1-B | 2 | TZISTR1198/TZISTR1152-19-1-1-1-B-B |  |
| 11 | | 4402 (/),4401-B/IITATZISTR1112-60-2-1-1-B | 3 | TZISTR1198/TZISTR1152-28-1-1-1-B-B |  |
| 12 | | 1133 LH59-N1-B/IITATZISTR1113-27-1-1-1-B | 4 | TZISTR1199/TZISTR1152-130-1-1-1-B-B |  |
|  | | Heat Stress tolerant lines derived from DTSTR BCs containing temperate germplasm |  | Selected lines susceptible to heat stress |  |
|  | | SET-2 |  | SET5 |  |
| 5 | | 1131(LH82-N1)-B/IITATZISTR1108-1-1-1-1-B | 17 | IITATZI1742/TZISTR1106-28-1-1-1-B | HB33-HB48 |
| 6 | | 1131(LH82-N1)-B/IITATZISTR1108-4-1-1-1-B | 18 | 1133 LH59-N1-B/IITATZISTR1110-8-1-1-1 |  |
| 7 | | 1131(LH82-N1)-B/IITATZISTR1108-28-1-1-1-B | 19 | 4402 (/),4401-B/IITATZISTR1112-9-5-1-1 |  |
| 8 | | 1133 LH59-N1-B/IITATZISTR1110-5-1-1-1-B | 20 | 1133 LH59-N1-B/IITATZISTR1113-14-1-1-1 |  |
|  | | Selected lines susceptible to heat stress |  | Heat Stress tolerant lines derived from DTSTR BCs containing temperate germplasm |  |
|  | | SET6 |  | SET3 |  |
| 21 | | 1131(LH82-N1)-B/IITATZISTR1108-19-1-1-1 | 9 | TZISTR1154/TZISTR1111-2-1-1-1-B | HB59-HB64 |
| 22 | | 1131(LH82-N1)-B/IITATZISTR1108-10-1-1-1 | 10 | TZISTR1154/TZISTR1111-34-1-2-1-B |  |
| 23 | | 1131(LH82-N1)-B/IITATZISTR1108-6-4-1-1 | 11 | TZISTR1198/TZISTR1152-140-1-1-1-B |  |
| 24 | | 1131(LH82-N1)-B/IITATZISTR1108-30-2-1-1 | 12 | TZISTR1198/TZISTR1152-162-1-2-1-B |  |
|  | | Selected lines susceptible to heat stress |  | Selected lines susceptible to heat stress |  |
|  | | SET4 |  | SET6 | HB65-HB80 |
| 13 | | 1131(LH82-N1)-B/IITATZISTR1108-19-1-1-1 | 21 | TZISTR1154/TZISTR1111-2-1-1-1-B |  |
| 14 | | 1131(LH82-N1)-B/IITATZISTR1108-10-1-1-1 | 22 | TZISTR1154/TZISTR1111-34-1-2-1-B |  |
| 15 | | 1131(LH82-N1)-B/IITATZISTR1108-6-4-1-1 | 23 | TZISTR1198/TZISTR1152-140-1-1-1-B |  |
| 16 | | 1131(LH82-N1)-B/IITATZISTR1108-30-2-1-1 | 24 | TZISTR1198/TZISTR1152-162-1-2-1-B |  |
|  | | Selected lines susceptible to heat stress |  | Selected lines susceptible to heat stress |  |
|  | | SET5 |  | SET-4 |  |
| 17 | | TZISTR1154/TZISTR1111-2-1-1-1-B | 13 | 1133 LH59-N1-B/IITATZISTR1110-60-1-1-1-B | HB81-HB96 |
| 18 | | TZISTR1154/TZISTR1111-34-1-2-1-B | 14 | 1133 LH59-N1-B/IITATZISTR1110-62-1-1-1-B |  |
| 19 | | TZISTR1198/TZISTR1152-140-1-1-1-B | 15 | 4402 (/),4401-B/IITATZISTR1112-60-2-1-1-B |  |
| 20 | | TZISTR1198/TZISTR1152-162-1-2-1-B | 16 | 1133 LH59-N1-B/IITATZISTR1113-27-1-1-1-B |  |
| Standard checks | | | | | |
| 25 | 9022-13 (Tolerant check) | |  |  | HB97 |
| 26 | 8338-1 (Susceptible check) | |  |  | HB98 |
| 27 | OBA SUPER 7 (Striga resistant commercial hybrid) | |  |  | HB99 |
| 28 | OBA SUPER 9 (Striga resistant commercial hybrid) | |  |  | HB100 |
