# Supplementary material for: Genetic analysis of tolerance to combined drought and heat stress in tropical maize
Source: PLoS One. 2024 Jun 20;19(6):e0302272. doi: 10.1371/journal.pone.0302272 (PMC11189248; doi:10.1371/journal.pone.0302272)
Supplement: S2 Table — Where YP, YP¯ grain yield and mean grain yield respectively under optimum condition and YS, YS grain yield and mean grain yield respectively under stress condition. (DOCX) [file pone.0302272.s002.docx]

| S. no. | Stress tolerance indices | Equations | References |
| --- | --- | --- | --- |
| 1 | Geometric mean productivity (GMP) | $\sqrt{Y_{P}x Y_{S}}$ | Schneider et al. (1997) stated that genotypes with larger GMP value will be more tolerant. |
| 2 | Mean productivity (MP) | $\frac{{Y_{P}+ Y}_{S}}{2}$ | Rosielle & Hamblin, (1981) explained genotypes with high value of MP will be more desired. |
| 3 | Harmonic mean (HM) | $\frac{{{2(Y}_{P}x Y}_{S})}{{Y_{P}+ Y}_{S}}$ | Jafari et al. (2009) revealed genotypes with high value of HM will be more acceptable. |
| 4 | Stress tolerance index (STI) | $\frac{{{( Y}_{P}x Y}_{S})}{\bar{Y}_{P}}$ | Schneider et al.(1997) indicated genotypes with high STI values will be tolerant to stress. |
| 5 | Tolerance index (TOL) | ${Y_{P}- Y}_{S}$ | Rosielle & Hamblin, (1981) showed genotypes with small TOL values more stable in stress conditions |
| 6 | Stress susceptibility index (SSI) | $\frac{1-\frac{Y_{S}}{Y_{P}}}{1-\frac{\bar{Y}_{S}}{\bar{Y}_{P}}}$ | Fischerab, (1978) told genotypes with small SSI values more stable in stress conditions |
| 7 | Yield stability Index (YSI) | $\frac{Y_{S}}{Y_{P}}$ | Bouslama & Schapaugh, (1984) the genotypes with high YSI values can be considered as stable genotypes under stress |
| 8 | Yield Index (YI) | $\frac{Y_{S}}{\bar{Y}_{S}}$ | Gavuzzi et al.(1997) exhibited genotypes with high value of YI will be suitable for stress condition. |

Supplementary Table 2. Equations and references of stress tolerance indices used to assess maize hybrids

Where$Y_{P}$, $\bar{Y}_{P}$ grain yield and mean grain yield respectively under optimum condition and$Y_{S}$, $\bar{Y}_{S}$ grain yield and mean grain yield respectively under stress condition
