# Supplementary material for: Genetic analysis of tolerance to combined drought and heat stress in tropical maize
Source: PLoS One. 2024 Jun 20;19(6):e0302272. doi: 10.1371/journal.pone.0302272 (PMC11189248; doi:10.1371/journal.pone.0302272)
Supplement: S3 Table — (DOCX) [file pone.0302272.s003.docx]

Supplementary Table 3. Mean squares from combined analyses for grain yield and other traits of 96 hybrids involving lines with varying resistance to tassel blast tested under combined drought and heat stress, managed drought stress and full irrigation for two years (2020 and 2021).

| Source of variation | DF | Husk cover (rate) | | | Plant aspect | | Anthesis silking interval | | |  |
| --- | --- | --- | --- | --- | --- | --- | --- | --- | --- | --- |
|  |  | CHDS | MDRTS | FIRR | CHDS | MDRTS | CHDS | MDRTS | FIRR |  |
| Year | 1 | 79*** | 0.29ns | 0.58* | 2.20*** | 0.01ns | 30*** | 0.94ns | 0.84* |  |
| Rep(Year) | 2 | 0.11ns | 0.01ns | 0.53*** | 0.08ns | 0.48* | 0.79ns | 0.13ns | 0.082ns |  |
| Block (Rep *Year) | 96 | 0.36ns | 0.23*** | 0.09ns | 0.29*** | 0.16ns | 0.77*** | 0.32ns | 0.21ns |  |
| Set | 5 | 0.78* | 0.14ns | 0.38*** | 0.87*** | 0.48*** | 0.53ns | 1.22*** | 0.54*** |  |
| Year*Set | 5 | 0.18ns | 0.07ns | 0.14ns | 0.14ns | 0.22ns | 0.51ns | 0.71ns | 0.13ns |  |
| Hybrid (Set) | 95 | 0.48*** | 0.12ns | 0.27*** | 0.40*** | 0.27*** | 72*** | 0.35ns | 0.23*** |  |
| Year*Hybrid (Set) | 95 | 0.27ns | .10ns | 0.14* | 0.25ns | 0.16ns | 0.74*** | 0.32ns | 0.14ns |  |
| Female (Set) | 18 | 0.77*** | 0.14ns | 0.31*** | 0.55*** | 0.34*** | 1.25*** | 0.38ns | 0.22ns |  |
| Male (Set) | 18 | 0.74*** | 0.09ns | 0.15ns | 0.53*** | 0.22* | 0.91*** | 0.30ns | 0.29* |  |
| Female*Male (Set) | 54 | 0.17ns | 0.12ns | 0.13ns | 0.17ns | 0.19* | 0.67* | 0.31ns | 0.18ns |  |
| Year*Female (Set) | 18 | 0.47* | 0.07ns | 0.11ns | 0.31* | 0.27* | 1.25*** | 0.33ns | 0.18ns |  |
| Year*Male (Set) | 18 | 0.34ns | 0.08ns | 0.09ns | 0.41*** | 0.22* | 0.90*** | 0.42ns | 0.12ns |  |
| Year*Female*Male(Set) | 54 | 0.17ns | 0.13ns | 0.09ns | 0.12ns | 0.12* | 0.71*** | 0.26ns | 0.10ns |  |
| R^2^ | | 0.90 | 0.83 | 0.86 | 0.89 | 0.81 | 0.89 | 0.77 | 0.79 |  |
| CV (%) | | 22.43 | 14.01 | 12.72 | 13.94 | 378.85 | 27.24 | 28.02 | 20.57 |  |

| Source of variation | DF | Ear per plant(EPP) | | | EPP transformed | Tassel blasting(TB) | TB transformed | Bare plant |
| --- | --- | --- | --- | --- | --- | --- | --- | --- |
|  |  | CHDS | MDRTS | FIRR | CHDS | CHDS | CHDS | CHDS |
| Year | 1 | 0.19*** | 0.02ns | 0.02ns | 9*** | 6*** | 3*** | 532*** |
| Rep(Year) | 2 | 0.09ns | 0.39*** | 0.04*** | 0.03ns | 0.05ns | 0.04ns | 2*** |
| Block (Rep *Year) | 96 | 0.06*** | 0.13*** | 0.01ns | 0.03*** | 0.43ns | 0.13ns | 0.65*** |
| Set | 5 | 0.04ns | 0.07ns | 0.002ns | 0.02ns | 0.94ns | 0.18ns | 0.52ns |
| Year*Set | 5 | 0.06ns | 0.06ns | 0.01ns | 0.02ns | 0.74ns | 0.15ns | 0.48ns |
| Hybrid (Set) | 95 | 0.08*** | 0.06ns | 0.01ns | 0.03*** | 0.64* | 0.12ns | 0.51*** |
| Year*Hybrid (Set) | 95 | 0.04ns | 0.05ns | 0.01ns | 0.02ns | 0.56ns | 0.11ns | 0.50*** |
| Female (Set) | 18 | 0.12*** | 0.06ns | 0.01ns | 0.07*** | 0.81* | 0.27*** | 0.45ns |
| Male (Set) | 18 | 0.12*** | 0.09*** | 0.01ns | 0.06*** | 0.57 | 0.17ns | 0.74*** |
| Female*Male (Set) | 54 | 0.03ns | 0.05ns | 0.01ns | 0.02ns | 0.43ns | 0.12ns | 0.34ns |
| Year*Female (Set) | 18 | 005* | 0.04ns | 0.003ns | 0.04* | 0.53ns | 0.16ns | 0.83*** |
| Year*Male (Set) | 18 | 0.05ns | 0.08* | 0.009ns | 0.03ns | 0.51ns | 0.15ns | 0.53* |
| Year*Female*Male(Set) | 54 | 0.03ns | 0.04ns | 0.006ns | 0.02ns | 0.49ns | 0.13ns | 0.31ns |
| R^2^ |  | 0.92 | 0.9 | 0.76 | 0.92 | 0.81 | 0.84 | 0.96 |
| CV (%) |  | 25.22 | 29.93 | 8.74 | 17.49 | 378.85 | 239.9 | 36.95 |

Supplementary Table 3. Continued …
