# Supplementary material for: Genetic analysis of tolerance to combined drought and heat stress in tropical maize
Source: PLoS One. 2024 Jun 20;19(6):e0302272. doi: 10.1371/journal.pone.0302272 (PMC11189248; doi:10.1371/journal.pone.0302272)
Supplement: S4 Table — (DOCX) [file pone.0302272.s004.docx]

Supplementary Table 4. SCA effect estimates for yield and yield related traits in 96 crosses produced through factorial mating of 24 inbred lines under combined heat and drought stress

| Code | GY | ANT | SIL | PH | PASP | EASP | Code | GY | ANT | SIL | PH | PASP | EASP |
| --- | --- | --- | --- | --- | --- | --- | --- | --- | --- | --- | --- | --- | --- |
| HB1 | -176.0 | 0.10 | 0.83 | 0.26 | -0.02 | 0.07 | HB29 | 47.91 | -0.09 | -0.05 | 0.10 | -0.01 | -0.01 |
| HB2 | 2.57 | 0.09 | 0.87 | 0.17 | -0.02 | 0.01 | HB30 | 21.54 | -0.03 | -0.07 | 2.02 | 0.00 | -0.03 |
| HB3 | 84.56 | -0.22 | 0.60 | 0.19 | 0.00 | -0.04 | HB31 | -116.6 | 0.12 | 0.13 | -0.95 | 0.01 | 0.00 |
| HB4 | 67.13 | 0.01 | 0.96 | -0.47 | -0.01 | -0.04 | HB32 | -45.21 | -0.05 | -0.05 | -2.65 | 0.03 | 0.07 |
| HB5 | -54.70 | 0.01 | 0.84 | 0.35 | -0.01 | 0.04 | HB33 | -7.88 | -0.04 | -0.07 | 1.80 | -0.04 | -0.07 |
| HB6 | 71.55 | -0.02 | 0.98 | 0.00 | 0.03 | -0.01 | HB34 | -157.9 | -0.11 | -0.13 | -0.71 | -0.01 | 0.08 |
| HB7 | -30.62 | 0.09 | 0.91 | -1.94 | 0.06 | 0.06 | HB35 | 87.8 | -0.11 | -0.03 | 0.84 | 0.01 | -0.03 |
| HB8 | -73.93 | 0.06 | 0.94 | 1.57 | -0.03 | -0.01 | HB36 | -127.7 | 0.42 | 0.39 | -1.88 | 0.07 | 0.13 |
| HB9 | 428.26 | -0.18 | 0.69 | -0.37 | -0.04 | -0.21 | HB37 | 76.1 | -0.17 | -0.11 | -1.15 | 0.03 | 0.03 |
| HB10 | -113.8 | 0.04 | 0.81 | 0.61 | 0.01 | 0.04 | HB38 | -113.3 | -0.11 | -0.02 | -1.34 | 0.07 | -0.01 |
| HB11 | -87.88 | -0.07 | 0.82 | 0.74 | -0.08 | 0.02 | HB39 | 87.41 | 0.19 | 0.11 | 0.23 | -0.05 | -0.10 |
| HB12 | -64.53 | 0.09 | 0.85 | -1.53 | 0.08 | 0.05 | HB40 | -20.38 | 0.12 | 0.05 | 0.77 | 0.00 | 0.09 |
| HB13 | -237.9 | 0.04 | 0.96 | -0.94 | 0.08 | 0.16 | HB41 | 255.67 | -0.21 | -0.15 | -0.36 | -0.05 | -0.15 |
| HB14 | -199.4 | 0.05 | 0.94 | -1.27 | 0.03 | 0.04 | HB42 | 195.22 | 0.17 | 0.12 | 1.07 | -0.05 | -0.05 |
| HB15 | -39.53 | 0.14 | 0.58 | -1.18 | -0.01 | -0.04 | HB43 | 87.65 | 0.07 | 0.02 | -0.87 | 0.02 | 0.02 |
| HB16 | 139.75 | -0.08 | 0.91 | -0.04 | -0.02 | -0.04 | HB44 | 109.8 | -0.37 | -0.30 | 0.06 | 0.01 | -0.15 |
| HB17 | 46.30 | 0.04 | -0.03 | 0.53 | 0.02 | 0.05 | HB45 | -173.8 | 0.37 | 0.29 | -0.07 | 0.03 | 0.14 |
| HB18 | 385.20 | -0.14 | -0.08 | -0.73 | -0.03 | -0.14 | HB46 | 90.80 | 0.07 | 0.04 | 1.55 | -0.02 | -0.07 |
| HB19 | 66.05 | -0.11 | -0.01 | -1.78 | 0.01 | -0.04 | HB47 | 235.6 | -0.32 | -0.24 | -0.31 | -0.02 | -0.07 |
| HB20 | -431.6 | 0.10 | 0.04 | 2.53 | 0.03 | 0.07 | HB48 | -284.2 | -0.01 | 0.03 | -0.76 | -0.01 | 0.09 |
| HB21 | 98.80 | -0.08 | -0.11 | -1.04 | 0.02 | -0.05 | HB49 | 32.19 | -0.05 | -0.06 | 0.19 | -0.05 | -0.01 |
| HB22 | -159.1 | 0.00 | 0.06 | 0.97 | -0.01 | 0.04 | HB50 | -306.3 | 0.07 | 0.01 | 0.54 | -0.01 | 0.11 |
| HB23 | -81.69 | 0.15 | 0.01 | 0.78 | 0.00 | 0.08 | HB51 | 288.4 | -0.06 | 0.00 | -0.46 | -0.01 | -0.03 |
| HB24 | -7.86 | 0.12 | 0.23 | -0.32 | 0.02 | 0.02 | HB52 | -1.52 | 0.10 | 0.14 | 0.63 | 0.02 | -0.01 |
| HB25 | -96.13 | 0.11 | 0.16 | 0.92 | -0.03 | -0.01 | HB53 | -138.7 | 0.20 | 0.13 | -1.49 | 0.04 | 0.03 |
| HB26 | -120.7 | 0.13 | 0.08 | -0.50 | 0.02 | 0.03 | HB54 | 99.31 | -0.05 | 0.01 | -1.75 | 0.01 | -0.07 |
| HB27 | -55.60 | -0.23 | -0.17 | -0.41 | 0.03 | 0.03 | HB55 | -87.87 | -0.03 | -0.04 | 0.90 | 0.02 | 0.05 |
| HB28 | 126.9 | -0.04 | -0.12 | -2.22 | 0.00 | 0.02 | HB56 | 405.36 | -0.16 | -0.15 | 4.21 | -0.09 | -0.12 |

Supplementary Table 4. Continued…

| Code | GY | ANT | SIL | PH | PASP | EASP | Code | GY | ANT | SIL | PH | PASP | EASP |
| --- | --- | --- | --- | --- | --- | --- | --- | --- | --- | --- | --- | --- | --- |
| HB57 | 115.93 | 0.03 | 0.00 | 0.66 | -0.04 | -0.09 | HB85 | 714.64 | -0.27 | -0.19 | 1.01 | -0.07 | -0.19 |
| HB58 | 77.93 | -0.19 | -0.13 | 0.86 | -0.01 | -0.04 | HB86 | 21.16 | 0.08 | 0.11 | 0.29 | -0.01 | 0.04 |
| HB59 | -137.8 | 0.01 | 0.03 | -0.52 | 0.06 | 0.05 | HB87 | -241.7 | -0.04 | -0.09 | 0.75 | 0.06 | 0.07 |
| HB60 | -164.8 | -0.02 | -0.01 | -1.48 | 0.04 | 0.08 | HB88 | -79.07 | 0.04 | 0.02 | -0.40 | -0.03 | 0.003 |
| HB61 | 170.15 | -0.36 | -0.21 | 1.74 | -0.02 | -0.10 | HB89 | -321.0 | 0.09 | 0.10 | -0.14 | 0.04 | 0.18 |
| HB62 | 362.37 | -0.02 | -0.06 | 1.11 | -0.04 | -0.10 | HB90 | 117.8 | -0.13 | -0.12 | 0.62 | -0.07 | 0.06 |
| HB63 | -172.9 | 0.18 | 0.10 | 0.11 | 0.00 | 0.06 | HB91 | 340.8 | -0.05 | -0.06 | 0.76 | -0.02 | -0.25 |
| HB64 | -645.1 | 0.24 | 0.18 | -4.44 | 0.08 | 0.22 | HB92 | -155.3 | 0.05 | 0.03 | 0.29 | 0.01 | -0.04 |
| HB65 | 382.33 | -0.30 | -0.15 | 2.72 | -0.05 | -0.24 | HB93 | -286.7 | -0.01 | -0.07 | 1.88 | 0.00 | 0.01 |
| HB66 | -28.34 | 0.01 | -0.08 | -0.31 | 0.04 | -0.001 | HB94 | -145.7 | 0.19 | 0.08 | -1.22 | 0.05 | 0.03 |
| HB67 | 73.86 | 0.02 | 0.00 | -0.49 | 0.00 | -0.04 | HB95 | 492.7 | -0.12 | -0.04 | -0.30 | -0.05 | -0.06 |
| HB68 | -71.27 | 0.10 | 0.09 | 0.36 | -0.09 | 0.09 | HB96 | 66.50 | -0.06 | -0.02 | 0.71 | 0.00 | 0.002 |
| HB69 | 142.92 | -0.10 | -0.06 | 0.24 | -0.02 | -0.13 |  |  |  |  |  |  |  |
| HB70 | 113.03 | -0.03 | -0.04 | 1.23 | -0.06 | 0.04 |  |  |  |  |  |  |  |
| HB71 | -151.3 | 0.16 | 0.04 | 0.47 | 0.03 | 0.07 |  |  |  |  |  |  |  |
| HB72 | 132.66 | -0.16 | -0.05 | 1.52 | -0.01 | -0.12 |  |  |  |  |  |  |  |
| HB73 | -260.0 | 0.32 | 0.13 | -2.99 | 0.04 | 0.20 |  |  |  |  |  |  |  |
| HB74 | 170.6 | -0.02 | -0.01 | 1.78 | -0.07 | -0.12 |  |  |  |  |  |  |  |
| HB75 | 47.15 | -0.11 | -0.06 | 1.22 | 0.00 | -0.04 |  |  |  |  |  |  |  |
| HB76 | -207.7 | 0.04 | 0.12 | -1.07 | 0.05 | 0.10 |  |  |  |  |  |  |  |
| HB77 | -226.9 | 0.14 | 0.07 | -0.06 | 0.04 | 0.19 |  |  |  |  |  |  |  |
| HB78 | -23.7 | -0.02 | 0.12 | -1.30 | 0.02 | 0.02 |  |  |  |  |  |  |  |
| HB79 | -36.87 | -0.12 | -0.01 | -0.13 | -0.04 | -0.04 |  |  |  |  |  |  |  |
| HB80 | -62.38 | 0.21 | -0.05 | -0.28 | 0.05 | 0.01 |  |  |  |  |  |  |  |
| HB81 | -296.1 | 0.27 | 0.20 | -1.46 | 0.05 | 0.07 |  |  |  |  |  |  |  |
| HB82 | 69.3 | -0.13 | -0.06 | 0.50 | 0.01 | -0.06 |  |  |  |  |  |  |  |
| HB83 | -41.70 | -0.12 | -0.08 | 0.30 | -0.04 | -0.03 |  |  |  |  |  |  |  |
| HB84 | 118.86 | 0.07 | 0.06 | 0.38 | 0.00 | 0.03 |  |  |  |  |  |  |  |
