# Supplementary material for: Genetic analysis of tolerance to combined drought and heat stress in tropical maize
Source: PLoS One. 2024 Jun 20;19(6):e0302272. doi: 10.1371/journal.pone.0302272 (PMC11189248; doi:10.1371/journal.pone.0302272)
Supplement: S5 Table — (DOCX) [file pone.0302272.s005.docx]

| Code | GY | ANT | SIL | PASP | Code | GY | ANT | SIL | PASP |
| --- | --- | --- | --- | --- | --- | --- | --- | --- | --- |
| HB1 | 222.1 | 0.71 | 0.72 | -0.07 | HB28 | -338.6 | -0.19 | -0.04 | 0.05 |
| HB2 | 245.3 | 0.34 | 0.18 | -0.16 | HB29 | 29.3 | -0.51 | -0.58 | -0.08 |
| HB3 | 235.5 | -0.90 | -0.89 | -0.05 | HB30 | -182.4 | -0.18 | 0.11 | -0.04 |
| HB4 | 295.3 | -0.66 | -0.49 | -0.14 | HB31 | -293.6 | 0.13 | 0.10 | 0.23* |
| HB5 | 273.9 | 0.37 | 0.42 | -0.09 | HB32 | -260.4 | 0.24 | 0.05 | 0.07 |
| HB6 | -142.8 | 0.31 | 0.32 | 0.12 | HB33 | 517.7* | 0.25 | 0.14 | -0.12 |
| HB7 | 221.3 | -1.03 | -0.98 | -0.02 | HB34 | -36.6 | -0.08 | 0.17 | -0.03 |
| HB8 | -448.4* | 0.46 | 0.54 | 0.17 | HB35 | -14.1 | -0.36 | -0.36 | 0.16 |
| HB9 | 117.1 | -0.10 | -0.45 | -0.002 | HB36 | -175.6 | 0.68 | 0.40 | 0.08 |
| HB10 | -32.2 | 0.23 | 0.29 | -0.04 | HB37 | 261.5 | 0.47 | 0.37 | -0.07 |
| HB11 | -113.3 | 0.64 | 0.70 | 0.02 | HB38 | 40.0 | -0.25 | -0.35 | 0.10 |
| HB12 | 119.5 | -0.07 | -0.08 | 0.03 | HB39 | -7.7 | 0.74 | 0.88 | -0.001 |
| HB13 | -231.6 | 0.17 | 0.28 | 0.05 | HB40 | -178.7 | 0.15 | 0.23 | 0.06 |
| HB14 | -275.2 | 0.004 | 0.09 | 0.17 | HB41 | 288.8 | -1.41* | -1.22 | -0.07 |
| HB15 | -426.6* | 1.32 | 0.98 | 0.11 | HB42 | -21.6 | 0.35 | 0.23 | -0.05 |
| HB16 | -59.5 | 0.37 | 0.19 | -0.06 | HB43 | 235.2 | -0.59 | -0.55 | -0.05 |
| HB17 | -39.8 | 0.16 | 0.11 | 0.14 | HB44 | 219.1 | -0.72 | -0.50 | -0.11 |
| HB18 | 432.7* | 0.36 | 0.37 | 0.14 | HB45 | -230.2 | 0.58 | 0.71 | 0.05 |
| HB19 | -368.9 | 0.66 | 0.82 | 0.15 | HB46 | -48.8 | 0.32 | 0.37 | -0.10 |
| HB20 | -380.0 | -0.01 | 0.06 | 0.14 | HB47 | 517.0* | -0.31 | -0.43 | -0.21 |
| HB21 | 322.2 | -1.20 | -0.95 | 0.14 | HB48 | -231.0 | 0.43 | 0.48 | 0.02 |
| HB22 | 232.4 | 0.39 | 0.16 | 0.15 | HB50 | 749.4*** | 1.47* | 1.39* | 0.41*** |
| HB23 | -186.9 | -0.61 | -0.55 | 0.15 | HB51 | -150.1 | 0.55 | 0.62 | 0.09 |
| HB24 | -250.6 | 1.18 | 1.07* | 0.14 | HB52 | -126.3 | 0.36 | 0.19 | -0.09 |
| HB25 | -103.1 | 1.19 | 1.23 | 0.10 | HB53 | 232.1 | -0.88 | -0.77 | -0.04 |
| HB26 | 20.4 | -0.84 | -0.88 | 0.04 | HB54 | -288.2 | 0.41 | 0.38 | 0.19 |
| HB27 | -105.9 | -1.03 | -1.11 | -0.07 | HB55 | 130.2 | -0.08 | 0.03 | -0.21 |

Supplementary Table 5. SCA effect estimates for yield and selected yield related traits in 96 single crosses produced through factorial mating of 24 inbred lines under managed drought stress

Supplementary Table 5. Continued …

| Code | GY | ANT | SIL | PASP | Code | GY | ANT | SIL | PASP |
| --- | --- | --- | --- | --- | --- | --- | --- | --- | --- |
| HB56 | 200.4 | 0.11 | 0.24 | 0.08 | HB83 | -124.3 | 0.20 | -0.02 | 0.03 |
| HB57 | 364.6 | -1.65* | -1.48* | -0.25* | HB84 | -68.2 | -0.15 | 0.24 | -0.08 |
| HB58 | 131.6 | -0.50 | -0.37 | 0.12 | HB85 | 205.6 | -0.60 | -0.54 | -0.07 |
| HB59 | -315.8 | 1.46* | 1.39* | 0.14 | HB86 | 9.2 | 0.38 | 0.29 | 0.03 |
| HB60 | -241.9 | -0.26 | -0.39 | 0.21 | HB87 | 88.9 | -0.76 | -0.71 | -0.01 |
| HB61 | 76.8 | -0.08 | -0.08 | 0.00 | HB88 | -242.4 | 0.41 | 0.42 | 0.00 |
| HB62 | 365.7 | -0.99 | -1.06 | -0.24 | HB89 | -43.9 | 0.24 | 0.15 | 0.05 |
| HB63 | -589.6* | 0.52 | 0.41 | 0.26* | HB90 | -289.3 | 0.23 | 0.27 | 0.12 |
| HB64 | -493.4* | 1.39 | 1.42* | 0.14 | HB91 | 408.0 | 1.45* | -1.15 | -0.18 |
| HB65 | 334.1 | -0.61 | -0.51 | 0.28* | HB92 | -7.8 | 0.77 | 0.48 | 0.17 |
| HB66 | -140.6 | 0.79 | 0.45 | 0.13 | HB93 | -264.8 | 0.41 | 0.51 | 0.10 |
| HB67 | 2.2 | 0.24 | 0.15 | -0.04 | HB94 | 617.7*** | 0.05 | 0.13 | -0.02 |
| HB68 | 391.4 | -0.85 | -0.71 | -0.15 | HB95 | 195.71 | -0.58 | -0.53 | -0.11 |
| HB69 | 749.4*** | 1.47* | 1.39* | 0.41*** | HB96 | -166.063 | -0.77 | -0.86 | 0.08 |
| HB70 | -150.1 | 0.55 | 0.62 | 0.09 |  |  |  |  |  |
| HB71 | -126.3 | 0.36 | 0.19 | -0.09 |  |  |  |  |  |
| HB72 | 232.1 | -0.88 | -0.77 | -0.04 |  |  |  |  |  |
| HB73 | -288.2 | 0.41 | 0.38 | 0.19 |  |  |  |  |  |
| HB74 | 130.2 | -0.08 | 0.03 | -0.21 |  |  |  |  |  |
| HB75 | 146.6 | -0.40 | -0.35 | 0.00 |  |  |  |  |  |
| HB76 | -355.0 | 1.09 | 0.95 | 0.10 |  |  |  |  |  |
| HB77 | -475.7* | 1.93* | 1.52* | 0.30* |  |  |  |  |  |
| HB78 | 58.7 | -1.29 | -1.01 | -0.05 |  |  |  |  |  |
| HB79 | 74.7 | 0.10 | 0.09 | -0.03 |  |  |  |  |  |
| HB80 | -210.7 | 0.30 | 0.20 | 0.02 |  |  |  |  |  |
| HB81 | -297.6 | 1.17 | 0.98 | 0.17 |  |  |  |  |  |
| HB82 | 100.4 | -0.47 | -0.64 | -0.09 |  |  |  |  |  |
