# Supplementary material for: Genetic analysis of tolerance to combined drought and heat stress in tropical maize
Source: PLoS One. 2024 Jun 20;19(6):e0302272. doi: 10.1371/journal.pone.0302272 (PMC11189248; doi:10.1371/journal.pone.0302272)
Supplement: S6 Table — (DOCX) [file pone.0302272.s006.docx]

Supplementary Table 6. SCA effect estimates for grain yield and selected yield related traits in 96 single crosses produced through factorial mating of 24 inbred lines under full irrigation

| Code | GY | ANT | SIL | PH | PASP | Female | GY | ANT | SIL | PH | PASP |
| --- | --- | --- | --- | --- | --- | --- | --- | --- | --- | --- | --- |
| HB1 | 203.4 | 0.19 | 0.20 | -1.57 | -0.01 | HB49 | -463.0 | -0.02 | 0.02 | 0.55 | 0.08 |
| HB2 | 82.1 | 0.30 | 0.36 | -0.10 | -0.01 | HB50 | 545.8 | 0.25 | 0.28 | -0.36 | -0.01 |
| HB3 | 228.8 | -0.39 | -0.49 | 1.07 | -0.05 | HB51 | 618.3* | -0.24 | -0.28 | 0.21 | -0.08 |
| HB4 | 317.3 | -0.20 | -0.19 | 1.25 | -0.05 | HB52 | 170.8 | 0.01 | 0.03 | 0.16 | -0.03 |
| HB5 | 434.4 | 0.11 | 0.15 | 0.84 | -0.09 | HB53 | -61.4 | 0.49 | 0.55 | -1.18 | 0.01 |
| HB6 | -38.3 | 0.04 | 0.01 | 1.02 | -0.001 | HB54 | -562.0 | -0.08 | -0.07 | -0.91 | 0.13 |
| HB7 | -227.9 | 0.03 | 0.11 | -0.18 | 0.02 | HB55 | 705.4* | -0.28 | -0.33 | 0.76 | -0.02 |
| HB8 | 554.4 | -0.35 | -0.44 | 0.39 | -0.13 | HB56 | 492.6 | -0.31 | -0.36 | 2.27 | -0.09 |
| HB9 | -1.8 | -0.11 | -0.03 | -0.015 | -0.03 | HB57 | -46.0 | -0.27 | -0.37 | 0.41 | -0.03 |
| HB10 | 102.0 | -0.07 | -0.04 | 0.61 | -0.09 | HB58 | 637.6* | -0.64 | -0.85 | 2.49 | -0.14 |
| HB11 | -112.8 | 0.11 | 0.06 | -0.58 | 0.06 | HB59 | -224.6 | 0.09 | 0.13 | -0.24 | 0.05 |
| HB12 | -511.3 | 0.34 | 0.33 | -1.13 | 0.12 | HB60 | -697.5 | 0.38 | 0.50 | -3.32 | 0.13 |
| HB13 | -224.6 | -0.11 | -0.19 | 0.26 | 0.06 | HB61 | 593.2 | -0.26 | -0.30 | 0.96 | -0.08 |
| HB14 | -383.0 | 0.09 | 0.18 | -1.62 | 0.02 | HB62 | 346.4 | -0.16 | -0.15 | 0.45 | -0.13 |
| HB15 | -33.4 | 0.15 | 0.17 | -1.39 | 0.04 | HB63 | -1057.2* | 0.65 | 0.78* | -1.06 | 0.15 |
| HB16 | -270.5 | 0.21 | 0.26 | -0.34 | 0.005 | HB64 | -631.7 | 0.13 | 0.11 | -0.78 | 0.09 |
| HB17 | 553.1 | 0.15 | 0.19 | 0.02 | -0.10 | HB65 | 423.5 | -0.05 | -0.10 | 0.75 | -0.06 |
| HB18 | 395.1 | -0.04 | 0.12 | 0.54 | 0.02 | HB66 | -746.7* | -0.07 | -0.01 | -1.37 | 0.10 |
| HB19 | -270.2 | -0.09 | -0.35 | -0.12 | 0.03 | HB67 | -245.1 | 0.27 | 0.18 | 0.59 | 0.03 |
| HB20 | -894.6* | 0.15 | 0.20 | -2.13 | 0.12 | HB68 | 278.3 | -0.23 | -0.22 | 1.11 | -0.03 |
| HB21 | -420.4 | -0.52 | -0.64 | -0.03 | 0.04 | HB69 | 472.2 | -0.52 | -0.37 | 2.12 | -0.08 |
| HB22 | 81.9 | 0.41 | 0.39 | 0.39 | -0.05 | HB70 | -307.3 | 0.41 | 0.53 | -0.36 | 0.05 |
| HB23 | -94.8 | -0.06 | 0.15 | -0.09 | 0.10 | HB71 | 421.4 | -0.06 | -0.37 | 1.21 | -0.06 |
| HB24 | -285.8 | 0.48 | 0.61 | -0.12 | 0.05 | HB72 | 251.9 | 0.48 | -0.43 | -0.70 | -0.06 |
| HB25 | -20.4 | 0.30 | 0.28 | -0.87 | 0.03 | HB73 | -761.2* | 0.30 | 0.52 | -1.98 | 0.17 |
| HB26 | -126.9 | -0.13 | -0.15 | -0.74 | 0.05 | HB74 | 59.1 | -0.13 | -0.13 | 1.51 | -0.03 |
| HB27 | -244.5 | 0.05 | 0.19 | -0.99 | 0.01 | HB75 | 242.7 | 0.05 | -0.33 | -0.68 | -0.13 |
| HB28 | 178.8 | -0.31 | -0.43 | 1.26 | 0.03 | HB76 | -255.5 | -0.31 | 0.29 | 0.18 | 0.03 |
| HB29 | 747.4* | -0.09 | -0.003 | 0.84 | -0.01 | HB77 | -612.4 | -0.09 | -0.02 | -1.69 | 0.07 |
| HB30 | 291.8 | -0.38 | -0.52 | 0.09 | -0.05 | HB78 | 470.8 | -0.38 | -0.44 | 0.52 | 0.00 |
| HB31 | -469.0 | 0.20 | 0.11 | -1.36 | 0.09 | HB79 | -120.4 | 0.20 | 0.33 | 0.06 | 0.08 |
| HB32 | -219.1 | 0.03 | 0.12 | -1.15 | -0.02 | HB80 | -220.6 | 0.03 | 0.38 | -0.28 | -0.01 |
| HB33 | 417.1 | -0.01 | -0.13 | -0.61 | -0.08 | HB81 | -127.5 | -0.11 | -0.18 | 0.04 | 0.00 |
| HB34 | -67.7 | 0.14 | 0.25 | 0.89 | 0.03 | HB82 | 192.3 | 0.06 | 0.23 | 0.52 | 0.07 |
| HB35 | 222.2 | -0.22 | -0.29 | -0.73 | -0.04 | HB83 | -485.0 | 0.31 | 0.32 | -1.87 | 0.05 |
| HB36 | -566.7 | 0.20 | 0.32 | -0.46 | 0.04 | HB84 | -76.1 | -0.06 | -0.14 | 1.70 | -0.07 |
| HB37 | -120.6 | 0.29 | 0.40 | 0.67 | -0.005 | HB85 | 235.5 | -0.01 | -0.02 | 0.04 | 0.03 |
| HB38 | 152.8 | -0.13 | -0.16 | -0.39 | -0.04 | HB86 | -510.1 | 0.32 | 0.53 | -1.36 | 0.14 |
| HB39 | -578.4 | 0.46 | 0.56 | -0.061 | 0.05 | HB87 | -215.4 | -0.41 | -0.56 | 0.80 | -0.02 |
| HB40 | 456.1 | -0.25 | -0.32 | 0.32 | 0.03 | HB88 | 7.3 | -0.04 | -0.07 | 1.07 | -0.04 |
| HB41 | 455.6 | -0.68 | -0.91* | -0.15 | 0.03 | HB89 | -134.4 | 0.31 | 0.32 | 0.19 | -0.03 |
| HB42 | 111.1 | 0.11 | 0.15 | 0.47 | -0.07 | HB90 | 253.9 | -0.42 | -0.73* | 1.94 | -0.09 |
| HB43 | 174.1 | -0.62 | -0.68 | 1.24 | -0.02 | HB91 | 209.5 | -0.21 | -0.27 | 0.35 | -0.07 |
| HB44 | 90.2 | 0.46 | 0.48 | -0.07 | -0.03 | HB92 | -84.4 | 0.29 | 0.46 | -1.33 | 0.10 |
| HB45 | 84.0 | 0.34 | 0.59 | 0.79 | -0.08 | HB93 | -31.4 | 0.02 | 0.12 | 0.002 | -0.08 |
| HB46 | 138.3 | -0.14 | -0.27 | 0.56 | -0.06 | HB94 | 174.8 | 0.21 | 0.16 | -0.68 | -0.01 |
| HB47 | 274.8 | 0.29 | 0.34 | -0.13 | -0.01 | HB95 | 733.3* | -0.40 | -0.46 | 2.53 | -0.10 |
| HB48 | -173.4 | -0.29 | -0.34 | -0.28 | 0.02 | HB96 | -250.3 | 0.09 | 0.09 | -1.44 | 0.005 |
