# Supplementary material for: Genetic analysis of tolerance to combined drought and heat stress in tropical maize
Source: PLoS One. 2024 Jun 20;19(6):e0302272. doi: 10.1371/journal.pone.0302272 (PMC11189248; doi:10.1371/journal.pone.0302272)
Supplement: S7 Table — (DOCX) [file pone.0302272.s007.docx]

| Genotype | GMP | | TOL | | MP | | HM | | SSI | | STI | | YI | | YSI | |
| --- | --- | --- | --- | --- | --- | --- | --- | --- | --- | --- | --- | --- | --- | --- | --- | --- |
|  | CHDS | DRTS | CHDS | DRTS | CHDS | DRTS | CHDS | DRTS | CHDS | DRTS | CHDS | DRTS | CHDS | DRTS | CHDS | DRTS |
| HB1 | 2602 | 3036 | 3587 | 3093 | 3160 | 3407 | 2142 | 2705 | 1.81 | 0.92 | 0.42 | 0.58 | 0.57 | 1.44 | 0.28 | 0.38 |
| HB2 | 2378 | 2559 | 2847 | 2634 | 2772 | 2878 | 2040 | 2275 | 1.70 | 0.93 | 0.35 | 0.41 | 0.56 | 1.21 | 0.32 | 0.37 |
| HB3 | 2252 | 2723 | 3462 | 2950 | 2841 | 3097 | 1786 | 2394 | 1.90 | 0.95 | 0.32 | 0.47 | 0.46 | 1.26 | 0.24 | 0.35 |
| HB4 | 4338 | 3003 | 654 | 2749 | 4350 | 3303 | 4326 | 2731 | 0.35 | 0.87 | 1.18 | 0.57 | 1.68 | 1.50 | 0.86 | 0.41 |
| HB5 | 2353 | 2864 | 3969 | 3443 | 3078 | 3342 | 1799 | 2455 | 1.96 | 1.00 | 0.35 | 0.51 | 0.46 | 1.26 | 0.22 | 0.32 |
| HB6 | 1352 | 1733 | 3443 | 3142 | 2189 | 2339 | 835 | 1284 | 2.21 | 1.19 | 0.11 | 0.19 | 0.19 | 0.60 | 0.12 | 0.20 |
| HB7 | 2640 | 2447 | 1984 | 2241 | 2820 | 2691 | 2471 | 2225 | 1.30 | 0.87 | 0.44 | 0.38 | 0.76 | 1.22 | 0.48 | 0.41 |
| HB8 | 1691 | 1532 | 4619 | 4718 | 2863 | 2813 | 999 | 834 | 2.24 | 1.35 | 0.18 | 0.15 | 0.23 | 0.35 | 0.11 | 0.09 |
| HB9 | 4000 | 2441 | -135 | 2418 | 4001 | 2724 | 4000 | 2188 | -0.09 | 0.91 | 1.00 | 0.37 | 1.70 | 1.18 | 1.03 | 0.39 |
| HB10 | 3531 | 2317 | 449 | 2336 | 3538 | 2595 | 3524 | 2069 | 0.30 | 0.92 | 0.78 | 0.34 | 1.38 | 1.11 | 0.88 | 0.38 |
| HB11 | 3338 | 1950 | -89 | 2139 | 3338 | 2224 | 3337 | 1710 | -0.07 | 0.96 | 0.70 | 0.24 | 1.41 | 0.90 | 1.03 | 0.35 |
| HB12 | 2877 | 2036 | -34 | 1411 | 2877 | 2154 | 2877 | 1923 | -0.03 | 0.73 | 0.52 | 0.26 | 1.21 | 1.12 | 1.01 | 0.51 |
| HB13 | 2227 | 1948 | 2454 | 2763 | 2543 | 2388 | 1950 | 1589 | 1.63 | 1.08 | 0.31 | 0.24 | 0.55 | 0.78 | 0.35 | 0.27 |
| HB14 | 1853 | 1451 | 1051 | 1594 | 1926 | 1655 | 1783 | 1271 | 1.07 | 0.96 | 0.22 | 0.13 | 0.58 | 0.67 | 0.57 | 0.35 |
| HB15 | 1521 | 1328 | 2697 | 2860 | 2033 | 1952 | 1138 | 904 | 2.00 | 1.25 | 0.15 | 0.11 | 0.29 | 0.40 | 0.20 | 0.15 |
| HB16 | 2092 | 1859 | 1716 | 2012 | 2261 | 2114 | 1936 | 1635 | 1.38 | 0.95 | 0.27 | 0.22 | 0.59 | 0.86 | 0.45 | 0.36 |
| HB17 | 3638 | 2488 | 2753 | 4090 | 3890 | 3221 | 3402 | 1922 | 1.31 | 1.15 | 0.83 | 0.39 | 1.05 | 0.91 | 0.48 | 0.22 |
| HB18 | 4308 | 3061 | 1046 | 2935 | 4340 | 3395 | 4277 | 2760 | 0.54 | 0.89 | 1.16 | 0.59 | 1.59 | 1.50 | 0.78 | 0.40 |
| HB19 | 1579 | 1023 | 1355 | 1959 | 1718 | 1416 | 1452 | 739 | 1.42 | 1.21 | 0.16 | 0.07 | 0.43 | 0.34 | 0.43 | 0.18 |
| HB20 | 1606 | 925 | 185 | 1197 | 1608 | 1102 | 1603 | 777 | 0.27 | 1.04 | 0.16 | 0.05 | 0.63 | 0.39 | 0.89 | 0.30 |
| HB21 | 2018 | 2426 | 2258 | 1731 | 2312 | 2576 | 1761 | 2285 | 1.64 | 0.74 | 0.26 | 0.37 | 0.49 | 1.33 | 0.34 | 0.50 |
| HB22 | 3404 | 2578 | 726 | 2031 | 3423 | 2771 | 3385 | 2398 | 0.48 | 0.79 | 0.73 | 0.42 | 1.28 | 1.36 | 0.81 | 0.46 |
| HB23 | 2444 | 1653 | 638 | 1802 | 2465 | 1882 | 2424 | 1451 | 0.57 | 0.96 | 0.37 | 0.17 | 0.89 | 0.76 | 0.77 | 0.35 |
| HB24 | 766 | 1379 | 2151 | 1602 | 1320 | 1595 | 445 | 1193 | 2.25 | 0.99 | 0.04 | 0.12 | 0.10 | 0.62 | 0.10 | 0.33 |
| HB25 | 2068 | 2142 | 3502 | 3432 | 2710 | 2745 | 1578 | 1672 | 1.97 | 1.14 | 0.27 | 0.29 | 0.40 | 0.80 | 0.21 | 0.23 |

Supplementary table 7. Stress tolerance indices of genotypes for drought and combined heat and drought stresses.

Supplementary table 7. Continued …

| Genotype | GMP | | TOL | | MP | | HM | | SSI | | STI | | YI | | YSI | |
| --- | --- | --- | --- | --- | --- | --- | --- | --- | --- | --- | --- | --- | --- | --- | --- | --- |
|  | CHDS | DRTS | CHDS | DRTS | CHDS | DRTS | CHDS | DRTS | CHDS | DRTS | CHDS | DRTS | CHDS | DRTS | CHDS | DRTS |
| HB26 | 2426 | 2235 | 2282 | 2515 | 2681 | 2564 | 2195 | 1948 | 1.50 | 0.97 | 0.37 | 0.31 | 0.64 | 1.01 | 0.40 | 0.34 |
| HB27 | 2254 | 1654 | 1079 | 1900 | 2318 | 1907 | 2192 | 1434 | 0.95 | 0.98 | 0.32 | 0.17 | 0.74 | 0.74 | 0.62 | 0.34 |
| HB28 | 1536 | 1303 | 2996 | 3177 | 2145 | 2055 | 1099 | 826 | 2.06 | 1.29 | 0.15 | 0.11 | 0.27 | 0.36 | 0.18 | 0.13 |
| HB29 | 3571 | 2826 | 3758 | 4563 | 4035 | 3632 | 3160 | 2199 | 1.59 | 1.14 | 0.80 | 0.50 | 0.90 | 1.05 | 0.36 | 0.23 |
| HB30 | 3641 | 2055 | 2390 | 4187 | 3832 | 2933 | 3459 | 1439 | 1.19 | 1.23 | 0.83 | 0.26 | 1.10 | 0.65 | 0.52 | 0.17 |
| HB31 | 2405 | 1348 | 778 | 2181 | 2436 | 1734 | 2374 | 1048 | 0.69 | 1.14 | 0.36 | 0.11 | 0.85 | 0.50 | 0.72 | 0.23 |
| HB32 | 1962 | 1607 | 1936 | 2337 | 2188 | 1987 | 1759 | 1299 | 1.54 | 1.09 | 0.24 | 0.16 | 0.51 | 0.63 | 0.39 | 0.26 |
| HB33 | 3741 | 3467 | 2343 | 2730 | 3920 | 3726 | 3570 | 3226 | 1.15 | 0.79 | 0.88 | 0.75 | 1.15 | 1.83 | 0.54 | 0.46 |
| HB34 | 2461 | 2251 | 2382 | 2635 | 2734 | 2608 | 2215 | 1942 | 1.52 | 0.99 | 0.38 | 0.32 | 0.64 | 1.00 | 0.39 | 0.33 |
| HB35 | 3466 | 2359 | 1643 | 3114 | 3562 | 2826 | 3373 | 1968 | 0.94 | 1.05 | 0.75 | 0.35 | 1.14 | 0.98 | 0.63 | 0.29 |
| HB36 | 1052 | 1670 | 2704 | 2156 | 1713 | 1987 | 646 | 1403 | 2.21 | 1.04 | 0.07 | 0.17 | 0.15 | 0.71 | 0.12 | 0.30 |
| HB37 | 3450 | 2647 | 1124 | 2332 | 3496 | 2892 | 3406 | 2422 | 0.69 | 0.85 | 0.75 | 0.44 | 1.22 | 1.34 | 0.72 | 0.43 |
| HB38 | 1347 | 2401 | 4011 | 3118 | 2416 | 2862 | 751 | 2014 | 2.27 | 1.04 | 0.11 | 0.36 | 0.17 | 1.01 | 0.09 | 0.29 |
| HB39 | 3625 | 2159 | -919 | 1736 | 3654 | 2327 | 3597 | 2003 | -0.72 | 0.80 | 0.83 | 0.29 | 1.72 | 1.13 | 1.29 | 0.46 |
| HB40 | 1571 | 2039 | 4025 | 3655 | 2553 | 2738 | 966 | 1518 | 2.21 | 1.18 | 0.15 | 0.26 | 0.23 | 0.71 | 0.12 | 0.20 |
| HB41 | 5505 | 3200 | -394 | 3384 | 5509 | 3620 | 5502 | 2829 | -0.19 | 0.94 | 1.90 | 0.64 | 2.38 | 1.50 | 1.07 | 0.36 |
| HB42 | 4233 | 2301 | 762 | 3488 | 4250 | 2887 | 4216 | 1834 | 0.41 | 1.11 | 1.12 | 0.33 | 1.61 | 0.89 | 0.84 | 0.25 |
| HB43 | 5033 | 2785 | -1094 | 2798 | 5062 | 3117 | 5003 | 2489 | -0.61 | 0.92 | 1.59 | 0.49 | 2.34 | 1.33 | 1.24 | 0.38 |
| HB44 | 3264 | 2496 | 1255 | 2375 | 3324 | 2764 | 3206 | 2254 | 0.80 | 0.89 | 0.67 | 0.39 | 1.12 | 1.22 | 0.68 | 0.40 |
| HB45 | 1218 | 2099 | 4559 | 3958 | 2584 | 2885 | 574 | 1527 | 2.35 | 1.20 | 0.09 | 0.28 | 0.13 | 0.70 | 0.06 | 0.19 |
| HB46 | 3610 | 2381 | 1431 | 3106 | 3680 | 2843 | 3541 | 1994 | 0.82 | 1.04 | 0.82 | 0.36 | 1.24 | 1.00 | 0.67 | 0.29 |
| HB47 | 4919 | 3225 | -891 | 2179 | 4939 | 3404 | 4898 | 3055 | -0.50 | 0.72 | 1.52 | 0.65 | 2.25 | 1.80 | 1.20 | 0.52 |
| HB48 | 1491 | 1578 | 3002 | 2928 | 2115 | 2152 | 1050 | 1156 | 2.08 | 1.20 | 0.14 | 0.16 | 0.26 | 0.53 | 0.17 | 0.19 |
| HB49 | 3193 | 1904 | 592 | 2466 | 3206 | 2269 | 3179 | 1599 | 0.42 | 1.04 | 0.64 | 0.23 | 1.21 | 0.80 | 0.83 | 0.30 |
| HB50 | 3554 | 3016 | 3663 | 4270 | 3999 | 3695 | 3160 | 2461 | 1.57 | 1.08 | 0.79 | 0.57 | 0.90 | 1.21 | 0.37 | 0.27 |

Supplementary table 7. Continued …

| Genotype | GMP | | TOL | | MP | | HM | | SSI | | STI | | YI | | YSI | |
| --- | --- | --- | --- | --- | --- | --- | --- | --- | --- | --- | --- | --- | --- | --- | --- | --- |
|  | CHDS | DRTS | CHDS | DRTS | CHDS | DRTS | CHDS | DRTS | CHDS | DRTS | CHDS | DRTS | CHDS | DRTS | CHDS | DRTS |
| HB51 | 3494 | 2923 | 2992 | 3683 | 3800 | 3455 | 3211 | 2474 | 1.42 | 1.03 | 0.79 | 0.57 | 0.96 | 1.25 | 0.44 | 0.30 |
| HB52 | 2845 | 2449 | 2296 | 2793 | 3068 | 2819 | 2638 | 2128 | 1.37 | 0.98 | 0.77 | 0.54 | 0.80 | 1.10 | 0.46 | 0.34 |
| HB53 | 3102 | 2057 | 1464 | 2840 | 3188 | 2500 | 3020 | 1693 | 0.94 | 1.07 | 0.51 | 0.38 | 1.02 | 0.84 | 0.63 | 0.28 |
| HB54 | 3582 | 1889 | 599 | 2978 | 3594 | 2405 | 3569 | 1483 | 0.39 | 1.13 | 0.60 | 0.27 | 1.37 | 0.71 | 0.85 | 0.24 |
| HB55 | 3632 | 3305 | 2881 | 3305 | 3907 | 3695 | 3376 | 2956 | 1.35 | 0.91 | 0.81 | 0.22 | 1.03 | 1.58 | 0.46 | 0.38 |
| HB56 | 4209 | 2708 | 1423 | 3507 | 4269 | 3227 | 4150 | 2273 | 0.72 | 1.04 | 0.83 | 0.69 | 1.48 | 1.14 | 0.71 | 0.30 |
| HB57 | 3887 | 2712 | -272 | 1794 | 3889 | 2856 | 3885 | 2574 | -0.18 | 0.71 | 1.11 | 0.46 | 1.68 | 1.52 | 1.07 | 0.52 |
| HB58 | 3329 | 2687 | 3099 | 3838 | 3672 | 3302 | 3018 | 2187 | 1.49 | 1.09 | 0.95 | 0.46 | 0.89 | 1.07 | 0.41 | 0.26 |
| HB59 | 1480 | 1654 | 2839 | 2683 | 2051 | 2129 | 1069 | 1284 | 2.05 | 1.14 | 0.70 | 0.45 | 0.26 | 0.61 | 0.18 | 0.23 |
| HB60 | 1827 | 1531 | 1088 | 1493 | 1907 | 1704 | 1752 | 1376 | 0.00 | 0.90 | 0.14 | 0.17 | 0.57 | 0.74 | 0.56 | 0.39 |
| HB61 | 4107 | 2546 | 1187 | 3377 | 4150 | 3055 | 4065 | 2122 | 0.63 | 1.05 | 0.21 | 0.15 | 1.48 | 1.06 | 0.75 | 0.29 |
| HB62 | 3713 | 2838 | 1947 | 3138 | 3838 | 3243 | 3591 | 2484 | 1.01 | 0.96 | 1.06 | 0.41 | 1.19 | 1.30 | 0.60 | 0.35 |
| HB63 | 1655 | 872 | 317 | 1403 | 1662 | 1119 | 1647 | 680 | 0.44 | 1.14 | 0.87 | 0.51 | 0.63 | 0.32 | 0.83 | 0.23 |
| HB64 | 1597 | 751 | 1436 | 2241 | 1751 | 1349 | 1457 | 419 | 1.46 | 1.34 | 0.17 | 0.05 | 0.43 | 0.18 | 0.42 | 0.09 |
| HB65 | 4107 | 2749 | -152 | 2157 | 4107 | 2953 | 4106 | 2559 | -0.09 | 0.79 | 0.16 | 0.04 | 1.74 | 1.45 | 1.04 | 0.47 |
| HB66 | 2346 | 1537 | -397 | 1061 | 2355 | 1626 | 2338 | 1453 | -0.46 | 0.73 | 1.06 | 0.47 | 1.06 | 0.85 | 1.18 | 0.51 |
| HB67 | 4013 | 2181 | -875 | 2278 | 4037 | 2460 | 3990 | 1933 | -0.61 | 0.93 | 0.35 | 0.15 | 1.87 | 1.02 | 1.24 | 0.37 |
| HB68 | 3863 | 2912 | 600 | 2143 | 3874 | 3103 | 3851 | 2733 | 0.36 | 0.76 | 1.01 | 0.30 | 1.49 | 1.58 | 0.86 | 0.49 |
| HB69 | 3933 | 3397 | 1605 | 2421 | 4014 | 3606 | 3853 | 3200 | 0.84 | 0.74 | 0.94 | 0.53 | 1.34 | 1.86 | 0.67 | 0.50 |
| HB70 | 3853 | 1937 | -189 | 2762 | 3855 | 2379 | 3852 | 1577 | -0.13 | 1.09 | 0.97 | 0.72 | 1.65 | 0.77 | 1.05 | 0.27 |
| HB71 | 2749 | 2484 | 4012 | 4268 | 3403 | 3275 | 2220 | 1884 | 1.86 | 1.17 | 0.93 | 0.24 | 0.58 | 0.89 | 0.26 | 0.21 |
| HB72 | 3993 | 2905 | 1931 | 3411 | 4108 | 3368 | 3881 | 2505 | 0.95 | 0.99 | 0.47 | 0.39 | 1.31 | 1.29 | 0.62 | 0.33 |
| HB73 | 754 | 1204 | 1799 | 1374 | 1174 | 1386 | 485 | 1046 | 2.17 | 0.98 | 1.00 | 0.53 | 0.11 | 0.54 | 0.13 | 0.34 |
| HB74 | 3499 | 2205 | -704 | 1627 | 3516 | 2351 | 3481 | 2069 | -0.56 | 0.76 | 0.04 | 0.09 | 1.61 | 1.19 | 1.22 | 0.49 |
| HB75 | 2919 | 2516 | 2014 | 2549 | 3088 | 2821 | 2760 | 2245 | 1.23 | 0.92 | 0.77 | 0.31 | 0.87 | 1.20 | 0.51 | 0.38 |

Supplementary table 7. Continued …

| Genotype | GMP | | TOL | | MP | | HM | | SSI | | STI | | YI | | YSI | |
| --- | --- | --- | --- | --- | --- | --- | --- | --- | --- | --- | --- | --- | --- | --- | --- | --- |
|  | CHDS | DRTS | CHDS | DRTS | CHDS | DRTS | CHDS | DRTS | CHDS | DRTS | CHDS | DRTS | CHDS | DRTS | CHDS | DRTS |
| HB76 | 1747 | 1587 | 2509 | 2666 | 2151 | 2072 | 1419 | 1215 | 1.85 | 1.16 | 0.19 | 0.16 | 0.37 | 0.57 | 0.26 | 0.22 |
| HB77 | 1151 | 1128 | 2043 | 2063 | 1539 | 1529 | 861 | 833 | 2.00 | 1.19 | 0.08 | 0.08 | 0.22 | 0.39 | 0.20 | 0.19 |
| HB78 | 3131 | 2263 | 1605 | 2765 | 3232 | 2652 | 3033 | 1931 | 1.00 | 1.01 | 0.62 | 0.32 | 1.01 | 0.98 | 0.60 | 0.31 |
| HB79 | 2872 | 2356 | 1697 | 2399 | 2994 | 2643 | 2754 | 2099 | 1.11 | 0.92 | 0.52 | 0.35 | 0.89 | 1.12 | 0.56 | 0.38 |
| HB80 | 1859 | 1719 | 2331 | 2480 | 2194 | 2120 | 1575 | 1394 | 1.74 | 1.09 | 0.22 | 0.19 | 0.43 | 0.68 | 0.31 | 0.26 |
| HB81 | 2302 | 1693 | 1684 | 2422 | 2451 | 2082 | 2162 | 1377 | 1.28 | 1.09 | 0.33 | 0.18 | 0.67 | 0.68 | 0.49 | 0.26 |
| HB82 | 2987 | 2468 | 2090 | 2763 | 3165 | 2828 | 2820 | 2153 | 1.24 | 0.97 | 0.56 | 0.38 | 0.88 | 1.12 | 0.50 | 0.34 |
| HB83 | 3562 | 1898 | -1057 | 1900 | 3601 | 2122 | 3523 | 1697 | -0.86 | 0.91 | 0.80 | 0.23 | 1.72 | 0.91 | 1.34 | 0.38 |
| HB84 | 1915 | 1817 | 1842 | 1962 | 2125 | 2065 | 1725 | 1599 | 1.52 | 0.95 | 0.23 | 0.21 | 0.50 | 0.84 | 0.40 | 0.36 |
| HB85 | 5044 | 2482 | -1912 | 2704 | 5134 | 2826 | 4956 | 2179 | -1.15 | 0.96 | 1.60 | 0.39 | 2.54 | 1.14 | 1.46 | 0.35 |
| HB86 | 3140 | 2048 | -208 | 1656 | 3141 | 2209 | 3138 | 1899 | -0.17 | 0.81 | 0.62 | 0.26 | 1.35 | 1.07 | 1.07 | 0.45 |
| HB87 | 3613 | 2283 | 48 | 2205 | 3613 | 2535 | 3613 | 2056 | 0.03 | 0.90 | 0.82 | 0.33 | 1.50 | 1.11 | 0.99 | 0.39 |
| HB88 | 2685 | 1703 | 1471 | 2695 | 2784 | 2171 | 2589 | 1335 | 1.05 | 1.13 | 0.45 | 0.18 | 0.85 | 0.64 | 0.58 | 0.23 |
| HB89 | 2871 | 2212 | 1852 | 2701 | 3016 | 2592 | 2732 | 1888 | 1.18 | 1.01 | 0.52 | 0.31 | 0.87 | 0.96 | 0.53 | 0.31 |
| HB90 | 2709 | 1849 | 3076 | 3917 | 3115 | 2694 | 2355 | 1270 | 1.66 | 1.24 | 0.46 | 0.21 | 0.66 | 0.57 | 0.34 | 0.16 |
| HB91 | 4879 | 3080 | -514 | 2579 | 4886 | 3339 | 4872 | 2841 | -0.28 | 0.82 | 1.49 | 0.60 | 2.14 | 1.59 | 1.11 | 0.44 |
| HB92 | 2965 | 2211 | 1418 | 2457 | 3049 | 2529 | 2884 | 1932 | 0.95 | 0.97 | 0.55 | 0.31 | 0.98 | 1.01 | 0.62 | 0.35 |
| HB93 | 1307 | 1786 | 3661 | 3297 | 2249 | 2431 | 759 | 1313 | 2.25 | 1.19 | 0.11 | 0.20 | 0.17 | 0.61 | 0.10 | 0.19 |
| HB94 | 3023 | 3221 | 2716 | 2451 | 3314 | 3446 | 2757 | 3010 | 1.46 | 0.77 | 0.57 | 0.65 | 0.82 | 1.72 | 0.42 | 0.48 |
| HB95 | 6048 | 2899 | -1270 | 3903 | 6081 | 3494 | 6015 | 2404 | -0.58 | 1.06 | 2.30 | 0.53 | 2.80 | 1.20 | 1.23 | 0.28 |
| HB96 | 2488 | 1834 | 1844 | 2634 | 2653 | 2258 | 2333 | 1490 | 1.29 | 1.09 | 0.39 | 0.21 | 0.72 | 0.73 | 0.48 | 0.26 |
| HB97 | 4519 | 2775 | 2783 | 4862 | 4728 | 3689 | 4319 | 2087 | 1.14 | 1.17 | 1.28 | 0.48 | 1.39 | 0.98 | 0.55 | 0.21 |
| HB98 | 3855 | 3096 | 3005 | 3940 | 4137 | 3670 | 3591 | 2613 | 1.34 | 1.03 | 0.93 | 0.60 | 1.10 | 1.32 | 0.47 | 0.30 |
| HB99 | 5301 | 3588 | -658 | 2397 | 5311 | 3783 | 5291 | 3404 | -0.33 | 0.71 | 1.76 | 0.81 | 2.35 | 2.01 | 1.13 | 0.52 |
| HB100 | 3332 | 2952 | 2215 | 2732 | 3512 | 3253 | 3162 | 2679 | 1.20 | 0.87 | 0.70 | 0.55 | 1.00 | 1.46 | 0.52 | 0.41 |
